# Isotopic investigation of skeletal remains at the Imdang tombs reveals high consumption of game birds and social stratification in ancient Korea

**DOI:** 10.1038/s41598-021-01798-y

**Published:** 2021-11-19

**Authors:** Kyungcheol Choy, Hee Young Yun, Seung Hee Kim, Sangsoo Jung, Benjamin T. Fuller, Dae Wook Kim

**Affiliations:** 1 Department of Cultural Anthropology, Hanyang University ERICA, Ansan, 15588 South Korea; 2 Department of Marine Sciences and Convergence Engineering, Hanyang University ERICA, Ansan, 15588 South Korea; 3 Daon Institute of Cultural Heritage, Daegu, 42265 South Korea; 4 Department of Archaeology and Heritage Studies, School of Culture and Society, Aarhus University, 8270 Højbjerg, Denmark; 5 Yeungnam University Museum, Yeungnam University, Daegu, 04763 South Korea

**Keywords:** Biogeochemistry, Environmental social sciences

## Abstract

Understanding the development of early states on the Korean Peninsula is an important topic in Korean archaeology. However, it is not clear how social structure was organized by these early states and what natural resources were utilized from their surrounding environments. To investigate dietary adaptation and social status in ancient Korea, stable isotope ratios and radiocarbon dates were measured from humans and animals from the Imdang cemetery, Gyeongsan city, South Korea. The results indicate that the Imdang diet was mainly based on C_3_ plants and terrestrial animals. Animal remains in the graves were directly consumed as daily food items as well as for ritual offerings. MixSIAR modeling results revealed that the dietary sources for the humans were: game birds > C_3_ plants > terrestrial herbivores > marine fish > C_4_ plants. The finding that the game birds represented the highest contribution to the whole diet, indicates that game birds must have been intensively hunted. Furthermore, elites consumed more game birds than their retainers and they also consumed seafood as a privileged dietary item in the Imdang society. This study demonstrates that the Apdok was a stratified society having high variations in the consumption of food items available to an individual and provides new insights about the subsistence and social status of the early ancient Apdok state on the Korean Peninsula.

## Introduction

The emergence of early ancient states and their territorial expansions on the Korean Peninsula is one of the most debated topics in Korean archaeology^1–6^. By the first century BC, the Korean Peninsula witnessed significant changes in social structure and economic strategies along with the introduction of iron technology^1,6,7^. Korean archaeologists refer to this transitional era as the Proto-Three Kingdom Period (BC 108–313 AD), characterized by the occurrence of local polities with intensive agriculture, an increase in iron production, and new pottery techniques^5,7^. As a result of the intensification of agriculture and the increased use of iron artefacts such as iron weapons and agricultural tools, many polities emerged on the floodplains near rivers of the Korean Peninsula^5,8,9^. It is suggested that the growth of many polities inevitably initiated competition for land and resources and consequently these internal conflicts between local polities resulted in the development of early states on the Korean Peninsula^8,9^. According to Korean historical records, only three or four main polities were fully developed into centralized states in Manchuria and the Korean Peninsula: *Goguryeo* in Manchuria and northern Korea, *Baekje* in the southwest, *Gaya* in the south, and *Silla* in the southeast^1,10–12^. For example, the Silla Kingdom began as one of 12 independent polities in the present-day north Gyeongsang area^11^ and it grew to become an ancient central state by conquering or merging with other neighboring polities, and eventually spread its territory over the southeastern part of the Korean Peninsula^11,13,14^.

Before the expansion of the Silla Kingdom over neighboring polities, there existed other small polities with distinctly local cultures during the Proto-Three Kingdoms period. According to one of the main Korean historical records, *Samguksagi* (The Histories of the Three Kingdoms written in 1145 AD), there was a local polity called ‘‘*Apdok*’’, an early ancient state that existed in the Gyeongsan Basin during this time period^15,16^. Among the neighboring states, Apdok developed as a local state and existed for several centuries adjacent to the early Silla Kingdoms^4,17,18^. However, there is little research on the subsistence activities and social structures of this early ancient state, even though the Apdok state is important for understanding the development of the early Korean states during the Proto-Three Kingdoms period (BC 108–313 AD). Nearly all previous studies on Apdok society relied on the archaeological characteristics of burial types and associated artifacts from the burials or earthen fortress surrounding the Imdang mounds^19–24^. In addition, there were scientific attempts to identify the kinship and family relationships among human remains from the Apdok burials using ancient mitochondrial DNA analysis^25,26^. Although these studies have made substantial contributions to increase our knowledge about the Apdok society, there is still a limited understanding of the subsistence activities and social status in this ancient state during the Proto-Three Kingdoms period.

**Figure 1:**
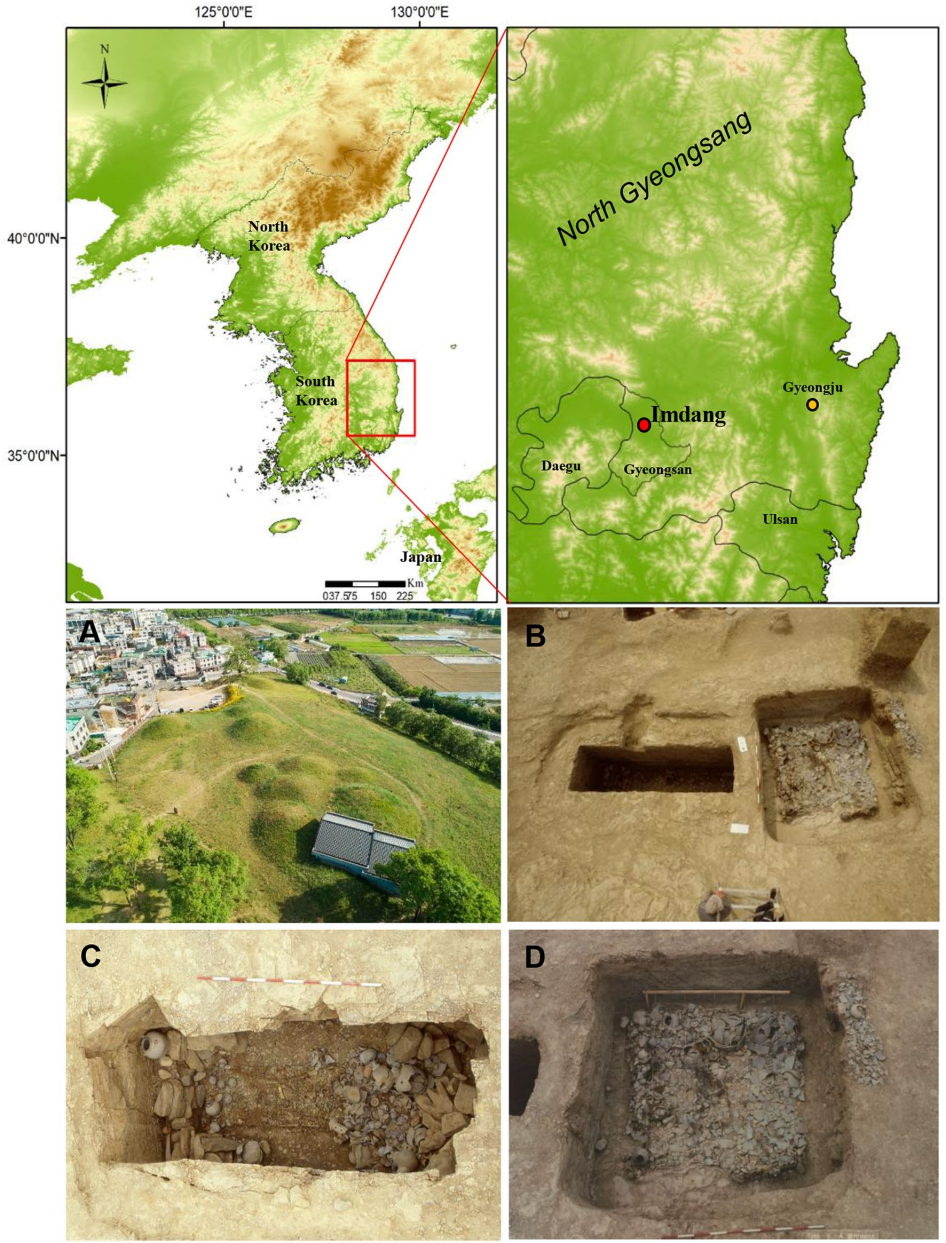
Map of the Korean Peninsula showing the location of the Imdang burials studied in this paper, and photos showing the site view of the Imdang burial mounds (**A**), exposure of a double chamber tomb after removing earthen mounds (**B**), the internal structure of a rectangle main chamber (**C**) and the other additional square chamber in a double chamber tomb (**D**) (Photos courtesy of YUM). Maps were created with software ArcGIS v 10.2 (https://www.esri.com/en-us/home).

Analysis of stable carbon and nitrogen isotope ratios is a useful tool for reconstructing palaeodiets in ancient populations^27,28^. Stable isotope analysis of associated human and animal remains can provide direct evidence of human diet and reflect the average isotopic composition of an individual’s intake of dietary proteins over long periods of time^29,30^. Furthermore, variation in stable isotopes can reveal social status within an ancient population^31,32^. While palaeodietary reconstruction using stable isotope analysis has been widely used, the application of isotope mixing models to archaeological materials has only recently increased in popularity^33,34^. Now there are several software packages that employ Bayesian mixing models for dietary reconstruction^34^. The MixSIAR model allows users to compare the estimated dietary compositions of two or more groups, provided all groups are directly comparable^35^. However, isotopic mixing models can be complicated for archaeological materials due to ecological differences between modern sources and prehistoric consumers^36^. To avoid the issue between modern and ancient ecosystems, in this study, we used consumers and food sources from the Imdang burial mounds and ran MixSIAR models to estimate the proportional contribution of contemporary food sources to each individual. The Imdang mound site is well known in South Korea for the elite burials associated with the early ancient state of Apdok located on the southeastern Korean Peninsula^16,37^ (Fig. 1). Fortunately, the Imdang site contains a large number of human and animal remains that were interred using different burial practices^16^. These human and animal remains from the Imdang burials provided valuable information for a wide range of human subsistence activities during the Proto-Three Kingdoms period. The aims of this study are to investigate the chronology of the different burials in the Imdang mounds through AMS radiocarbon dates and determine the dietary patterns of the Imdang population using the MixSIAR model. Through this, we attempt to reconstruct the subsistence practices of the Apdok society and examine if social stratification present in the burial practices was also reflected in food consumption.

## Archaeological context

The Imdang burial mounds are a well-known archaeological site located on a hillside of a mountain in the middle of an alluvial plain that was formed near Gyeongsan City, South Korea^4,38^ (see Fig. 1). The burial site contained approximately 1600 burials and a large amount of grave goods such as gilt-bronze crowns and ornaments, pottery, iron weapons and tools as well as human and animal bones (Supplementary Information). The large number of burial mounds that contain different types of burials suggests the possibility of multiple burials. According to the archaeological reports, three different types of burials: single wooden chambers (*dankwakmyo*), double wooden chambers (*jubukwakmyo*), and stone chambers with a horizontal entrance (*hoenggusik suksilmyo*) were identified at the Imdang burial mounds^20^. Among the reported 182 graves, the double wooden chambers (*jubukwakmyo*) are the most common. The double chamber tombs were mainly comprised of a main rectangle (*jukwak*) and a square auxiliary chamber (*bukwak*)^20^ (Fig. 1). During the excavations, the skeletal remains of 259 individuals within 182 graves were recovered and examined by the Yeungnam University Museum (YUM)^39,40^.

Analysis of the archaeobotanical remains showed that domesticated plants were recovered in the Imdang graves. Rice grains (*Oryza sativa*) with husks were found on the floor of the chamber and inside pottery, and marks of rice were found on the surface of iron artifacts inside burials^41^. Along with rice, foxtail millet (*Setaria italica*), barnyard millet (*Echinochloa crusgalli*), and perilla (*Perilla frutescens*) were also recovered from the burials^41^. Seeds of fruits such as peach (*Prunus persica*), Caucasian persimmon (*Diospyros lotus* L.), and apricot (*Prunus armeniaca*) were recovered in container jars^41^. The Imdang burials contained a variety of terrestrial and marine animals. A large variety of bird bones were exposed, and non-migratory birds such as pheasant (*Phasianus colchicus*), and migratory birds such as wild goose (*Anser*), swan (*Cygnus*), bustard (*Otis*), crane (*Grus*) and mallard (*Anas*) were frequently identified^42^ (Supplementary Fig. 1). In addition, more than 69 terrestrial mammals were found^43,44^. This included diverse specimens of wild herbivores such as deer (*Cervus*), wild boar (*Sus scrofa*), and hare (*Lepus*). Along with wild animals, domestic animals such as dogs (*Canis familiaris*), pigs (*Sus domesticus*), cattle (*Bos taurus*), and horses (*Equus caballus*) were found on the capstone and stone slabs of the graves^43^. Although located on an alluvial plain about 60 km from the coast, the Imdang burials contained a variety of marine fish and shellfish^41,45,46^ (Supplementary Fig. 1). The marine fish species from the Imdang burials were sharks (*Carcharhinidae*, *Lamnidae*, *Squalidae*), amberjack (*Seriola*), sea breams (*Sparidae*), rockfish (*Sebastiscus*), flatfish (*Paralichthyidae*), and puffer fish (*Tetraodontidae*)^41^. Imdang burials also exposed a large amount of shells of several species of clams, sea snails, abalones and scallops^41,45^.

## Results

To examine the integrity of the extracted collagen, the atomic C:N, % carbon and % nitrogen were calculated. Of the humans (n = 52) and animals (n = 22) analyzed, 22 human and 8 animal bones failed to produce enough collagen with yields below 1% and/or had C: N outside the accepted range of 2.9–3.6^47,48^. Thus, a total of 30 humans and 14 animals are reported in this study (Supplementary Tables 2 and 3).

### Radiocarbon dating

The radiocarbon dating results for the human and faunal collagen from the Imdang burial mounds are listed in Supplementary Table 1. The 10 human and 2 animal bones from each burial were measured and the dates were calibrated by the CALIB REV8.2 program^49^. According to the dating results, the Imdang humans ranged in age from approximately 80 BC to 394 cal AD (Supplementary Table 1). Thus, the Imdang cemetery was used for over 400 years, and all the burials belong to the Proto-Three Kingdom (108 BC–313 AD) and the initial phase of the Three-Kingdom (313–668 AD) period. For the animals, the radiocarbon date of the dog is 295 AD, which is similar to the human dates, but the median age of the sandbar shark (393 BC) is much older than the humans due to the marine reserve effect^50,51^.

### Fauna

**Figure 2:**
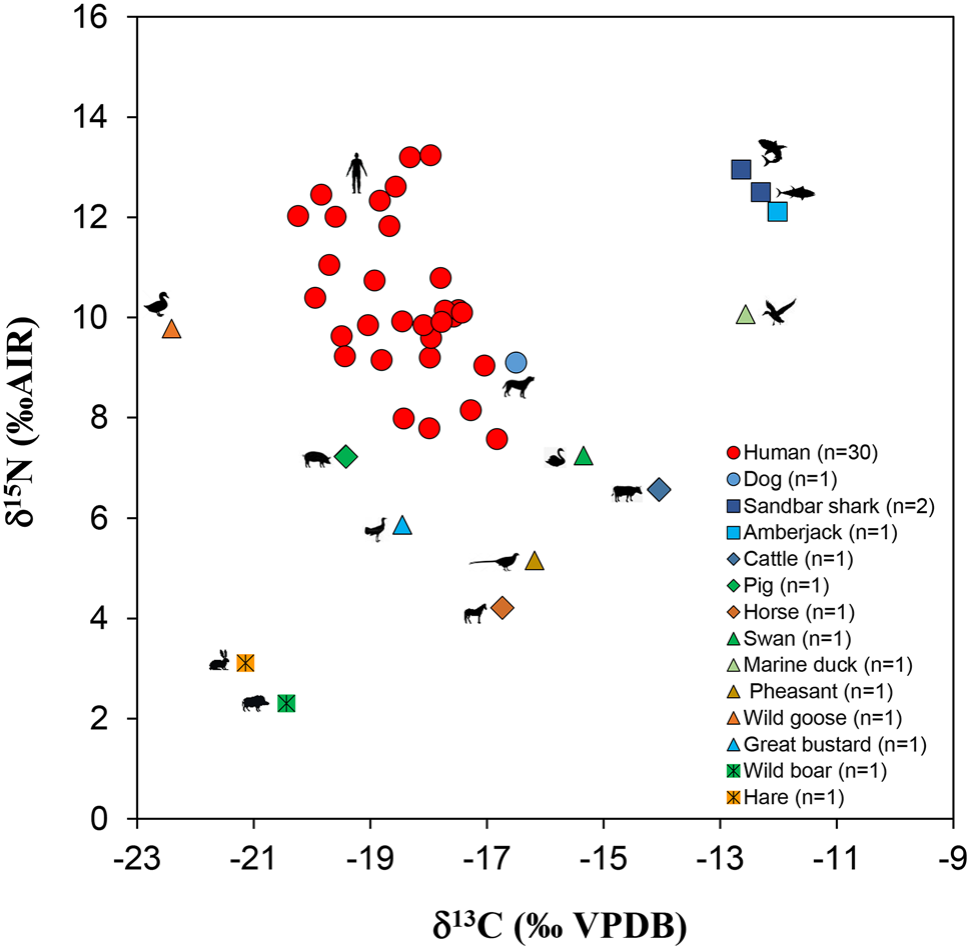
Bulk δ^13^C and δ^15^N values of human and fauna remains from Joyeung E (I, II, III) burials in this study.

Animal bone collagen isotopic results are presented in Fig. 2 and Supplementary Table 2. The analysis of the faunal samples from the Imdang burials provides baseline isotopic data against which the human δ^13^C and δ^15^N values will be compared. The hare (*Lepus*) and wild boar (*Sus scrofa*) had isotopic results within the range of C_3_ plant consumers (average ± SD, δ^13^C = − 20.8 ± 0.5‰ and δ^15^N = 2.7 ± 0.6‰). The carbon isotopic results from these terrestrial herbivores indicate a predominance of C_3_ vegetation in this region. The isotopic results from the five game birds indicate that the pheasant (*Phasianus*), bustard (*Otis*), goose (*Anser*) and swan (*Cygnus*) have C_3_ terrestrial signatures (average ± SD, δ^13^C = − 18.1 ± 3.2‰ and δ^15^N = 7.0 ± 2.0‰) and the other, duck (*Anas*) has a marine signature with δ^13^C = − 12.6‰ and δ^15^N = 10.1‰. The mean δ^13^C and δ^15^N values from the two sandbar sharks (*Carcharhinus*) were δ^13^C = − 12.6‰ and δ^15^N = 12.4‰, and the mean values from the amberjack fish (*Seriola*) is δ^13^C = − 12.0‰ and δ^15^N = 12.1‰. The isotopic results of these marine fish are similar to those of previous published marine animals (shark, tuna fish, dolphin) from archaeological sites in South Korea^52–54^.

In contrast to the wild herbivores, the horses (*Equus caballus*) and cattle (*Bos taurus*) have higher δ^13^C and δ^15^N values (mean δ^13^C = − 15.4 ± 1.9 ‰ and δ^15^N = 5.4 ± 1.7‰). The δ^13^C value of the livestock indicates that they consumed a substantial proportion of C_4_ plants in their diet, likely millet or other C_4_ grasses fed to them by humans. A single pig (*Sus domesticus*) has a lower δ^13^C value (− 17.0‰) and a higher δ^15^N (7.2‰) than the horse and cattle. This reflects an omnivorous diet consisting primarily of human food leftovers/waste. A single dog (*Canis familiaris*) has δ^13^C = − 16.5‰ and δ^15^N = 9.1‰ which is similar to the humans and suggests possible consumption of human foods^55^ (Fig. 2).

### Humans

Human collagen isotopic results are presented in and Fig. 2 and Supplementary Table 3. The δ^13^C values range from − 20.2 to − 16.2‰, with a mean ± SD value of − 18.1 ± 0.9‰. The δ^13^C values reflect that the majority of the humans were consuming C_3_ terrestrial resources. However, the wide range of δ^13^C values (4.0‰) indicates that the Imdang humans also consumed additional resources with elevated δ^13^C values, such as marine foods and millet. The δ^15^N values of the individuals range from 7.6 to 13.3‰, with a mean ± SD value of 10.3 ± 1.5‰. There is approximately a 3–5‰ shift in the mean δ^15^N values between the humans and the game birds (δ^15^N: 7.0 ± 2.0‰), and livestock (δ^15^N: 5.1 ± 2.3‰), indicating that the humans likely consumed a significant amount of these animals for dietary protein.

### Isotopic differences and social status

**Figure 3:**
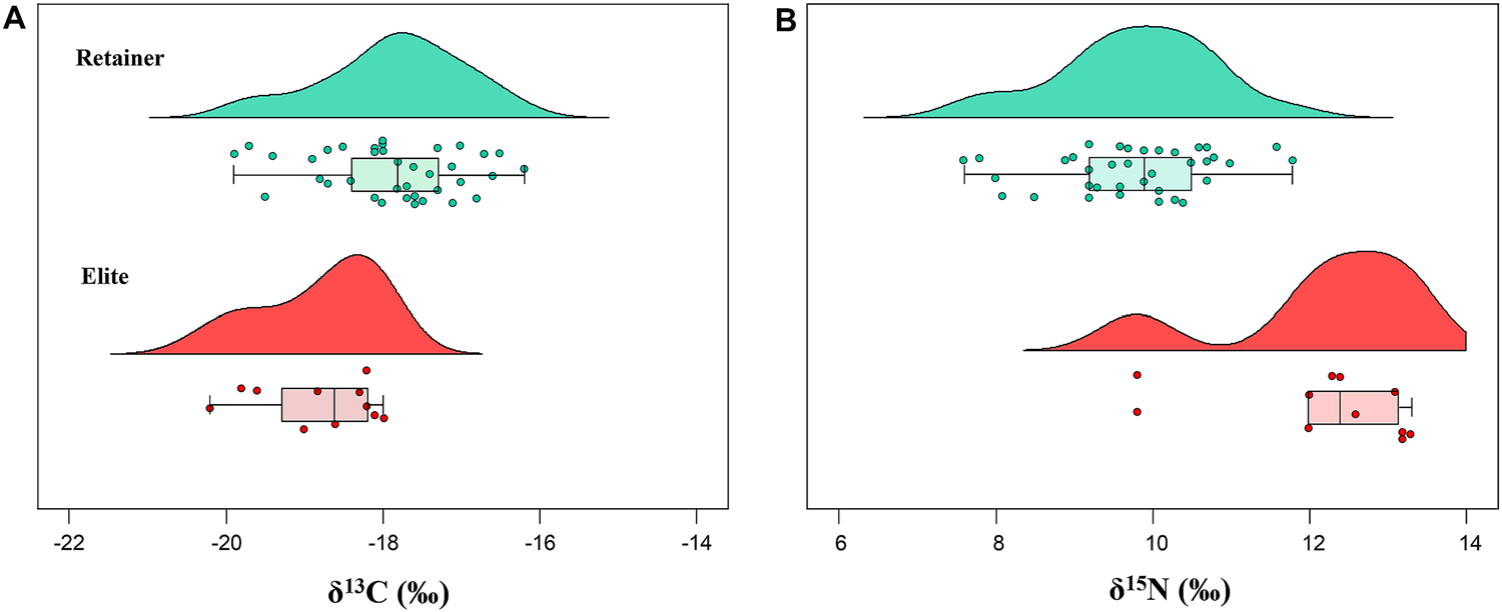
Raincloud plots showing the stable carbon (**A**) and nitrogen (**B**) isotope values between elites and retainers from the Imdang burial mounds. There are significant differences in both δ^13^C (*P* < 0.0001) and δ^15^N (*P* < 0.0001) values between elites and retainers.

Based on archaeological features and burial structures, the Joyeung district was divided into Joyeung C (I, II) and E (I, II, III). In this study, humans (n = 30) from Joyeung E were measured, but humans (n = 18) from Joyeung C were previously published^56^. To investigate the relationship between the isotopic results and the social status of the Imdang individuals, humans from the Joyeung C district are included here for discussion. According to the archaeological and isotope data, all 48 individuals were categorized into either elite (n = 12) or retainer (n = 33) groups. A simple t-test found that there is a significant difference in the δ^15^N values between the elites and retainers (δ^15^N: t = 6.6, *P* < 0.0001). The mean δ^15^N value for the elites is 2.5‰ higher than the retainers (elite: 12.2 ± 1.3‰ > retainer: 9.7 ± 1.0‰). This likely indicates that the elites consumed more animal protein than their retainers. Furthermore, there is a significant difference in δ^13^C values between the elites and the retainers (δ^13^C: t = − 3.8, *P* < 0.0001) (Fig. 3). The mean δ^13^C value for the elites is 1.0‰ lower than those of the retainers (elite: − 18.8 ± 0.8‰ < retainer: − 17.8 ± 0.8‰). These results suggest that the elites consumed less C_4_ plants than their retainers. These isotopic data demnonstrate that there is a significant difference in diets according to the social class (*elite vs. retainer*), even though both were buried in similar wooden chamber tombs.

### Isotopic differences associated with burial type, sex and age

Among the 29 burials, 24 belong to the double wooden chamber tombs, which are composed of two chambers: one main rectangle (*jukwak*) and the other square auxiliary (*bukwak*) chamber (Fig. 1). It is believed that the main chamber was constructed for elites, while the auxiliary chamber was constructed for retainers. Isotopic difference between the main (n = 28) and auxiliary chamber (n = 15) were examined. A t-test found no significant differences in the isotopic values of the humans between the main and auxiliary chambers in the Imdang mounds (δ^13^C: t = − 0.88, *P* = 0.38; δ^15^N: t = 1.33, *P* = 0.19). This indicates that there is no isotopic difference between the main and auxiliary chambers, suggesting that type and shape of the chambers are associated with the quantity of grave goods, not associated with social status.

Relationships between the isotopic results and the sex of the individual, age groups and burial types were also investigated. Among the 48 individuals, 12 males and 9 females were identified based on sex. No statistically significant difference in the isotopic values between the males and females was found (δ^13^C: t = − 0.86, *P* = 0.40; δ^15^N: t = 1.8, *P* = 0.08). This indicates that there was no detectable difference in food consumption between the males and females. To examine the possibility of age-related isotopic differences, a total of 48 individuals were grouped into children (0–20 years) and adults (> 20 years) following the age–at-death determined by the YUM^40^. The δ^13^C values exhibit no significant difference between the children and adults (t = 1.04, *P* = 0.30). However, there is a statistically significant difference in δ^15^N values (t = − 3.25, *P* < 0.002) between the children and the adults. The mean δ^15^N value for the adults is 1.3‰ higher than those of the children (adult: 10.7 ± 1.4‰ > child: 9.4 ± 0.9‰). This difference in δ^15^N values suggests that the consumption of animal proteins increased as individuals became older.

### MixSIAR model results

**Figure 4:**
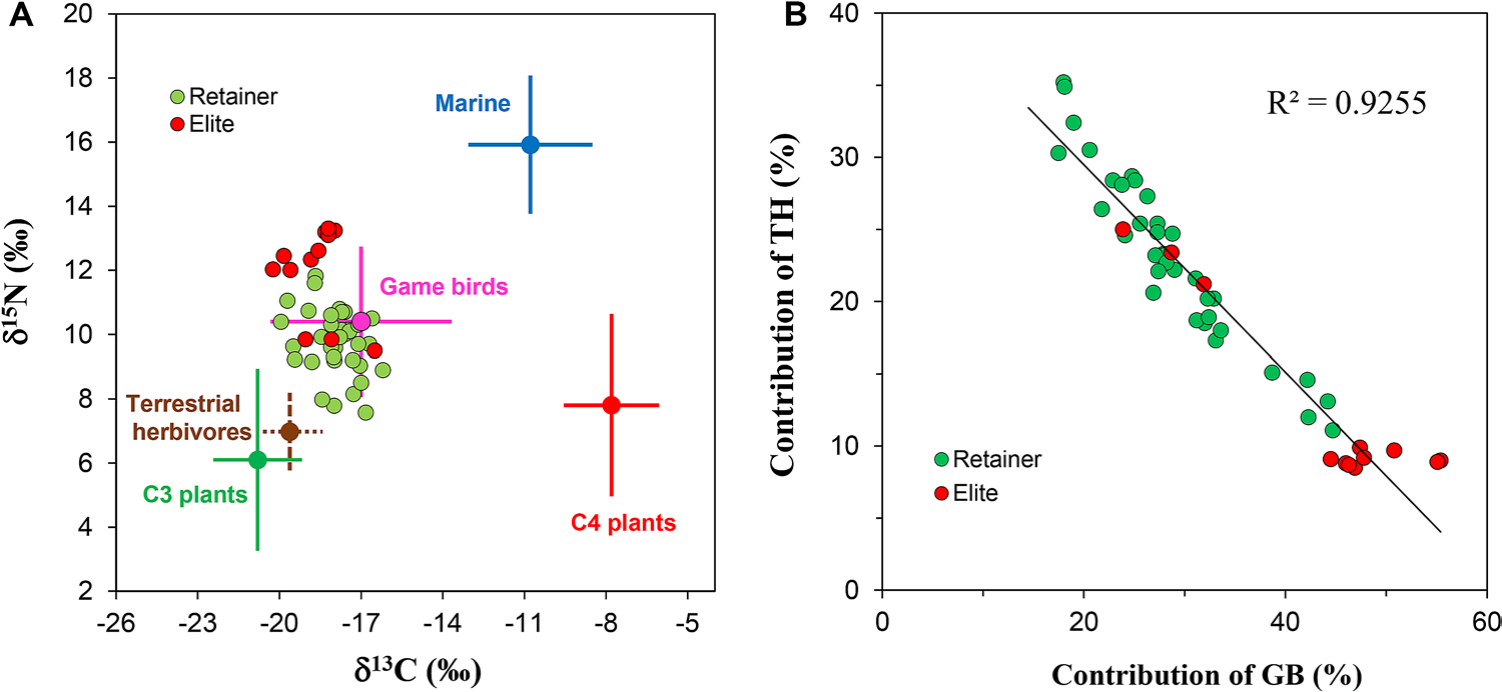
Isotopic plot of humans from the Imdang tombs in relation to the potential five food sources generated by the MixSIAR model (**A**) and the relationship between the percent contributions of the game birds (GB) and terrestrial herbivores (TH) to each human (**B**). The previously published isotopic values of humans (n = 18) from Joyeung C^56^ are added and calculated using the MixSIAR model. Isotopic values of the five food sources were adjusted for trophic discrimination factors (C_3_ plant: Δ^13^C = 5.2‰ and Δ^15^N = 3.8‰; C_4_ plant: Δ^13^C = 4.5‰ and Δ^15^N = 3.8‰; animal: Δ^13^C = 1‰ and Δ^15^N = 3.8‰)^33,34,69^. In terms of contributions for each food to the human diet, there was a negative correlation between game birds and terrestrial herbivores (r = − 0.962, *P* < 0.0001).

**Figure 5:**
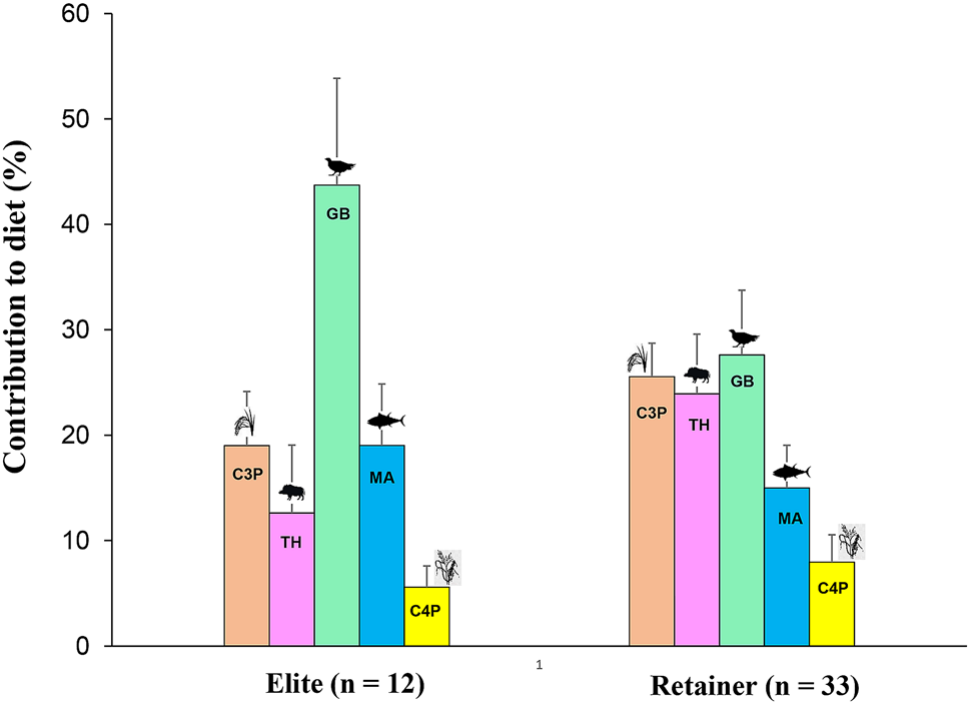
A bar chart representation of the proportional contribution of the five food groups (C_3_ plants, terrestrial herbivores, game birds, marine animals, C_4_ plants) to the Imdang humans. Elites show a higher contribution of game birds (GB) and marine animals (MA), while retainers have a higher contribution of terrestrial herbivores (TH), C_3_ plants (C3P), and C_4_ plants (C4P).

In order to investigate isotopic differences based on food groups, the δ^13^C and δ^15^N values of the Imdang humans were compared using univariate ANOVA analysis. The δ^13^C and δ^15^N values between the Imdang humans and the five different food groups were investigated (C_3_ plants, terrestrial herbivores, game birds, marine fish, C_4_ plants). Significant differences in the δ^13^C values (F_(239.6)_, df = 5, *P* < 0.0001) and δ^15^N values (F_(114.4)_, df = 5, *P* < 0.0001) between humans and the five food groups were found. The Imdang humans had higher δ^15^N values than the four food groups, except for the marine fish. The Imdang humans had lower δ^13^C values than three food groups (game birds, marine animals, C_4_ plants) except for the C_3_ plants and terrestrial herbivores (Fig. 4). The MixSIAR model was used to estimate the proportional contribution of these five food sources to the humans. The model outputs revealed that the Imdang humans had a wide range of contributions from the game birds (18–55%), C_3_ plants (14–33%), terrestrial herbivores (8–35%), marine fish (8–27%), and C_4_ plants (5–15%) (Supplementary Table 4). These results reveal that the dietary sources for the humans were as follows: game birds > C_3_ plants > terrestrial herbivores > marine fish > C_4_ plants. In addition, there was a strong positive correlation between the game birds and marine animals (r = 0.406, *P* < 0.004), and a negative correlation between game birds and terrestrial herbivores (r = − 0.962, *P* < 0.0001) (Fig. 4). There is a significant difference in food consumption between individuals even in a single burial. For instance, in the wooden chamber tomb of Joyeung E-1-2ho (Supplementary Fig. 3), individuals (SID-05) with higher δ^15^N values (≥ 12.0‰) have the highest contribution of both game birds (49%) and marine animals (21%), while individuals (SID-04) with lower δ^15^N values (≤ 9.0‰) have the highest contribution of both terrestrial herbivores (31%) and C_3_ plants (27%). Thus, there must have been very different food consumption patterns within the same community. This is especially true for the elites which had a higher contribution of game birds (44%) and marine animals (19%), while the retainers had a higher contribution of game birds (28%), C_3_ plants (26%) and terrestrial herbivores (24%) (Fig. 5).

## Discussion

Although several attempts have been made to construct a chronology based on burial structure and associated pottery^4,20,23^, there is no absolute internal chronology of the burials at the Imdang mounds. The radiocarbon dates prove that the Imdang cemetery was used from approximately 80 BC to 394 AD (Supplementary Fig. 2). This new chronology indicates that the Imdang cemetery belongs to the Proto-Three Kingdoms period and the initial phase of the Three-Kingdoms period. This finding compares with the previous radiocarbon dating results of six wooden pillars excavated from the Imdang burials^57^. The AMS radiocarbon dates from the wooden pillars showed that the results (94–135 AD and 224–289 AD) were within the same range of the radiocarbon dates of the human collagen. However, the AMS dates presented here are not consistent with the existing chronologies based on the pottery typology. Pottery chronology suggested that double wooden chambers appeared one century later than our radiocarbon dates of human bones^23^. This discrepancy comes from methodological difference between radiometric and relative dating, but there is no difference in the chronological sequences of the main burial practices.

In addition, the radiocarbon dates provide interesting insights into the burial practices and show how the burial traditions have changed over time. The radiocarbon dates indicate that there is a chronological sequence among the three types of burials. The earliest burial types are single wooden chambers (*dankwakmyo*), which appeared around 80 BC and lasted until the first century AD. It is believed that the first use of single wooden chambers as tombs originated from the nomadic tribes of the steppes in southern Manchuria, and this new burial system with horse sacrifice and iron armor ultimately diffused to the southern part of the Korean Peninsula^3,4,58^. The single wooden chambers were replaced by double wooden chambers (*jubukwakmyo*) around the late second century AD, and then double wooden chambers became a main burial type of the Imdang cemetery until the late fourth century AD. Basically, the double wooden chambers were of the same style as the single wooden chambers, but larger with two chambers to allow more grave goods and offerings, and larger mounds were constructed above the graves^37^. Our radiocarbon dates show that the double wooden chamber tombs at Imdang were the main burial type for more than 200 years. The square stone chambers with a horizontal entrance (*hoenggusik suksilmyo*) also appeared at least around the end of the third century but these did not become the main burial type at the Imdang society.

The results from the MixSIAR modeling found that game birds (33%), C_3_ plants (24%) and terrestrial herbivores (20%) were the main food sources for the Imdang population, while marine animals (16%) and C_4_ plants (7%) were also additionally consumed. This indicates that the majority of the dietary protein (84%) at the Imdang site was from terrestrial sources. The results also indicate that the Imdang inhabitants depended more on C_3_ plants (rice, wheat, barley and soybean) than C_4_ plants (millets). In particular, the contribution of C_3_ plants (24%) to the total diet is three times higher than C_4_ plants (7%). In addition, the results also show that animal protein had a large proportional contribution (69%) to the whole diet. Within the five food groups, game birds were the highest contribution to the whole diet of the Imdang people, indicating the intensive hunting of game birds (pheasants, bustard, wild goose, and swan) (Fig. 5). Terrestrial herbivores also had a significant contribution to the whole diet of the Imdang population. The Imdang people hunted wild herbivores (deer, wild boar and hare) for meat. Overall, most animal foods in the Imdang diets derived from the subsistence hunting of game birds, and terrestrial herbivores.

The work presented here is significant to better understanding the subsistence economy during the Proto-Three Kingdoms period (BC 108–313 AD). Previous isotopic studies indicated that humans had diets mainly based on C_3_ plants and terrestrial animals during the Proto-Three Kingdoms^56,59^. However, it is difficult to infer which food items were consumed because flora and fauna remains were not found with the burials. In this study, our data showed that C_3_ plants (rice, peach, persimmon, and apricot) and terrestrial animals (pheasant, bustard, wild boar, deer, and hare) were the main resources in the diet of Imdang. This means that domesticated plants as well as hunting wild animals played an important role in the Imdang economy. Furthermore, our data show that marine sources (sharks, amberjack, sea breams, rockfish, and puffer fish) made a detectable contribution (16%) to the Imdang diet. This suggests that the inland Imdang people intentionally imported and consumed marine fish and shellfish from the coastal regions. Thus, we can assume that the Apdok had a diversified subsistence strategy for stable food supply. Several studies have suggested that humans domesticated and consumed the livestock during this time period^59^. However, it is not possible to determine how much livestock were consumed due to their isotopic similarity to the game birds. Thus, additional work is needed to better determine the significance of livestock to the total subsistence economy during the Proto-Three Kingdoms period.

Past zooarchaeological analysis found that numerous remains of terrestrial and marine animals were recovered from the Imdang tombs^37,60^. Although many of these skeletal remains were recovered from the main and auxiliary chambers, it is not clear whether these terrestrial and marine animals were directly consumed or only used for funeral ceremonies. Previous studies suggested that the animals in the chambers were processed for consumption^60^. Most pheasants in the double wooden chambers were recovered without heads, necks, feet, and wings and the disarticulated remains of only meaty parts were found in container jars^12,37^. It is believed that the animals were a specialty for mortuary rituals and funeral ceremonies. However, our isotopic results clarify that the terrestrial and marine animals found in the Imdang mounds were eaten for daily food items as well as used for ritual offerings. In particular, game birds represented a significant protein source in the Imdang daily diet.

In the Imdang tombs, no differences in diets were found based on sex, age or burial type. However, there were significant differences in the diets between elites and retainers, even though they were buried in the same graves. This suggests that the social position of individuals was much more important than the sex and age of an individual. Thus, we can postulate that the isotopic variation among the individuals reflects their different social positions within the Apdok society. The model outputs show that the Imdang elites consumed greater amounts of game birds and seafood. In particular, the elites had a higher contribution of seafood to the whole diet compared with the retainers. Thus the Imdang elites consumed more marine foods, even though there was an additional procured expense to transport the marine fish and shell fish from the coast. In contrast, the retainers had a higher contribution of plant resources to their diet compared to the elites. The retainers consumed more C_4_ plants than the elites, suggesting a limited access to animal resources and more consumption of millets. The isotopic variation within individuals supports the notion that the Imdang was a stratified society with a strict system of food supply and demand.

Archaeological evidence demonstrates that the Imdang was a stratified society. The luxurious grave goods, the scale of tombs and the practice of human sacrifice indicate there were social differences in the Imdang population. The large amount of labor needed for construction and the exceptional quantity and quality of the luxurious goods indicate that the tomb’s owners might have been the highest-ranking elites in Imdang society. In addition, archaeological evidence indicates that multiple sacrificed individuals (*sunjang*) were buried with a tomb’s owner and the practice of human sacrifice existed in the Imdang population until the introduction of Buddhism^12^. Retainer sacrifice was performed before the burial of the chamber and the construction of the tomb’s mound. Even though most of all the sacrificed individuals had a lower social position than the tomb’s owner, some of the sacrificed individuals interred in the auxiliary chamber were wearing luxurious ornaments for the elite class, indicating perhaps that they may have been at a level not far below the highest-ranking elites^37^. Through this, we can guess that the Imdang society had a deliberate social hierarchy system, which contained more than two social statuses.

Historical records also suggest that there were social status differences among the population during the Proto-Three Kingdoms^15,16^. Silla, one of the main neighboring states next to Apdok, had a well-known social system called the “bone rank (*golpumje*)” system^15,61^. The social system of the Silla classified members of the officials based on eight classes according to their lineage and it regulated not only the official grade and intermarriage but also the size and style of housing, furniture, food and clothing among the classes. Even though no direct historical evidence about the social structures of the Apdok exist, we can postulate that the Apdok also had a social hierarchy system like the neighboring Silla, which regulated the construction of tombs and the items of grave goods at the funerals of the elites. However, it is not clear whether the social stratification of the Apdok was mainly developed from their own culture or built in response to the influence of the Silla. In this study, the isotopic results clearly show that the Apdok had a hierarchic society where there was a great deal of variation in food consumption among individuals. While this research represents an important step to better understand the social structure and food economy in an early state on the Korean Peninsula, more isotopic research is needed to strengthen and further test the interpretations made here.

## Methods

Human and animal samples were selected from the Imdang collections at the Yeungnam University Museum in Daegu, South Korea. The Imdang site is divided into the three different burial districts (Joyeung, Imdang, and Bujeok). Human and animal bones analyzed in this study derived from the Joyeung and Imdang districts. Bone sampling was conducted following the identification of human and animals undertaken by the YUM^40,41,44^. All the fauna studied here were from the burials. A total of 52 humans and 22 animal bones are chosen for stable isotope ratio analysis.

### Radiocarbon dating

A total of 10 human and 2 animal bones (Supplementary Table 1) from each burial type were selected for radiocarbon dating because the extracts from these individuals met the quality criteria for collagen (%C, %N and C:N). The collagen was dated at the Keck Carbon Cycle AMS Facility at the University of California Irvine, USA.

### Sample preparation and collagen extraction

Collagen extraction for carbon and nitrogen stable isotope ratio analyses was undertaken according to the protocols outlined in Richards and Hedges^62^ with the addition of an ultrafiltration step^63^ at the Department of Archaeology, Aarhus University in Denmark. Bone samples (500 mg) were cut and cleaned with deionized water and dried for 3 days in ambient temperature. Dried samples were demineralized in 0.5 M HCl at 4 °C. Demineralized bones were then rinsed in deionized water and gelatinzed at 70 °C in a pH 3 solution for 48 h. The insoluble fraction was then filtered, first with 5 mm Ezee filters (Elkay Laboratory Products) and then the remaining solution was ultra-filtered to collect purified collagen using > 30 kDa filters by centrifugation at 2500 rpm. The purified solution was then frozen and freeze dried for 48 h. In this study, we did not extract lipids from human and animal bone samples due to the low contents, as measured by their C:N ratios, and the small amount of waterlogged bone samples made the influence of the lipid content negligible.

### Measurement of bulk stable carbon and nitrogen isotopes

The dried collagen was placed in tin foil capsules and analyzed for carbon and nitrogen isotope ratios at the Alaska Stable Isotope Facility (ASIF) in the University of Alaska Fairbanks by using continuous-flow isotope ratio mass spectrometers. A Costech ECS4010 Elemental Analyzer (Costech Scientific Inc, Valencia, CA) combusted samples to carbon dioxide and nitrogen gas, which were carried in a constant flow of helium to a Finnigan Delta Plus XP isotope ratio mass spectrometer via the Conflo III interface (Thermo-Finnigan Inc, Bremen, Germany). Data are presented in the accepted delta notation as δX = (R sample − R standard)/(R standard) × 1000‰, where R is the ratio of heavy to light isotope (for both nitrogen and carbon) and the internationally recognized standards are atmospheric nitrogen and Vienna Pee Dee Belemnite for carbon. Multiple peptone standards (δ^13^C = − 15.8‰, δ^15^N = 7.0‰) were concurrently run to assess analytical accuracy and precision. The carbon and nitrogen isotope values were calculated relative to the Vienna Pee Dee Belemnite (VPDB) for δ^13^C and atmospheric N_2_ (AIR) for δ^15^N, respectively. Replicate measurement errors on known standards were less than ± 0.2‰ for both δ^13^C and δ^15^N.

### Statistical analysis

Here t-tests as a test of differences in means among burial types, age groups and sexes were used. Normality and homogeneity of variances were checked by means of Levene’s and Shapiro–Wilk tests, respectively. Pearson correlations were used for assessing the presence of significant relationships among the considered variables. Potential food sources can be grouped according to ecological, archaeological and isotopic relevance. Food resources were categorized into five food groups: terrestrial herbivores (i.e., deer, wild boar, hare), marine (i.e., sandbar shark, bream, sea mullet, amberjack), game birds (i.e., pheasants, bustard, wild goose, swan), C_3_ (i.e., rice, wheat, soybean), and C_4_ (foxtail millet, broomcorn millet, barnyard millet) plants. In this study, we were not able to separate the livestock (horse, dog, cattle, pig) from the game birds due to their isotopic similarity. Thus, we combined the livestock with the game birds according to isotopic relevance. Isotopic data of three food groups (game birds, marine animals, terrestrial herbivores) derived from the Imdang burials and other archaeological sites in South Korea (Nukdo, Tongsamdong, Ando, Dongnae)^52–54,59^ and two plant groups from archaeological and modern isotopic data in South Korea and East Asia were used^64–68^. We used univariate analysis of variance (ANOVA) to evaluate possible differences in isotopic values among the five food groups (C_3_ plant, marine animal, game birds, terrestrial herbivores, C4 plant). A post hoc Turkey’ multiple test was used to evaluate differences between each food group and the Imdang humans. A MixSIAR model was used to estimate the proportional contribution of each food group to each individual, providing an estimate of the variability in the estimated proportion^35^. We incorporated the δ^13^C and δ^15^N values of the humans, the five food groups as well as trophic discrimination factors (C_3_ plant: Δ^13^C = 5.2‰ and Δ^15^N = 3.8‰; C_4_ plant: Δ^13^C = 4.5‰ and Δ^15^N = 3.8‰; animal: Δ^13^C = 1‰ and Δ^15^N = 3.8‰) to predict accurately the dietary proportions^33,34,69^. The level of significance was set at *P* ≤ 0.05. All statistical tests were performed using an R statistical program.

## Supplementary Information

Supplementary Information 1.

Supplementary Information 2.

Supplementary Information 3.

Supplementary Information 4.

Supplementary Information 5.

Supplementary Information 6.
