# Supplementary material for: Isotopic investigation of skeletal remains at the Imdang tombs reveals high consumption of game birds and social stratification in ancient Korea
Source: Sci Rep. 2021 Nov 19;11:22551. doi: 10.1038/s41598-021-01798-y (PMC8605008; doi:10.1038/s41598-021-01798-y)
Supplement: Supplementary file 4 — Supplementary Information 4. [file 41598_2021_1798_MOESM4_ESM.docx]

**Supplementary Information**

**Isotopic investigation of skeletal remains at the Imdang tombs reveals high consumption of game birds and social stratification in ancient Korea**

Kyungcheol Choy^1^*, Hee Young Yun^2^, Seung Hee Kim^2^, Sangsoo Jung^3^, Benjamin T. Fuller^4^, Dae Wook Kim^3^

*^1^Department of Cultural Anthropology, Hanyang University ERICA, Ansan, South Korea, 15588*

*^2^Department of Marine Sciences and Convergence Engineering, Hanyang University ERICA, Ansan, South Korea, 15588*

*^3^Yeungnam University Museum, Yeungnam University, Daegu, South Korea, 04763*

*^4^Department of Archaeology and Heritage Studies, School of Culture and Society, Aarhus University, Højbjerg, DK-8270, Denmark*

**1. Stable isotope ratio analysis and palaeodietary reconstruction**

Stable isotope ratio analysis of bone collagen is used to reconstruct the protein portion of diets in ancient populations^1,2^. This approach has allowed the investigation of subsistence activities in ancient society, in particular, their relationship to social status within a population^3-5^. The two most common stable isotope ratios used for palaeodiets are carbon and nitrogen. In a terrestrial ecosystem the ratio of stable carbon isotopes is determined by the particular photosynthetic pathway of a plant. The two primary photosynthetic pathways used in isotopic research are known as C_3_ and C_4_. C_3_ plants have δ^13^C values between -20‰ and -34‰, and C_4_ plants have δ^13^C ranging from -9‰ to -16‰^6,7^. Consequently, C_3_ plant consumers have very different carbon isotope compositions from those of C_4_ plant consumers^8^. Rice (C_3_) and millet (C_4_) were prominent crops in the development of agriculture in East Asia. The δ^13^C values of modern foxtail millet (*Setaria italica*) and modern rice (*Oryza sativa*) are, on average, -11.8‰

and -26‰, respectively ^9-11^. Thus, it is possible to discriminate between the consumption of rice and millet in this region.

Nitrogen stable isotope ratios reflect trophic position within an ecosystem, since body tissues show a 3–5‰ elevation in δ^15^N values relative to consumed foods with each trophic level increase^12,13^. Thus, human δ^15^N values higher than associated faunal δ^15^N values are interpreted as indicating a diet with significant quantities of these animals, whereas δ^15^N values close to the associated herbivore values are interpreted as indicating a diet based mainly on plant proteins^14^. The combined use of nitrogen and carbon stable isotope ratios is also used as an indicator of the consumption of marine foods in the diet^1,3^. Humans that obtain the majority of their protein from marine foods have δ^13^C values close to -12‰, and δ^15^N values between 12 and 22‰, while individuals that consume only terrestrial protein sources have δ^13^C values of -20‰, and δ^15^N values ranging from 5 to 12‰^15^. Humans that had a mixture of marine and terrestrial protein would have isotope values somewhere between those end points^3^.

While palaeodietary reconstruction using stable isotope analysis has been widely used, the application of isotope mixing models to archaeological materials has only recently increased in popularity^16-18^. Using isotopic data from consumers and sources, isotopic mixing model can estimate the relative contribution of each source to the consumer’s diet. The approach of using stable isotopic measurements to quantify dietary compositions was first applied in ecological studies using the basic concepts of isotopic mass balance^19,20^. Now there are several software packages that employ Bayesian mixing models for dietary reconstruction^18^. The two most commonly used in archaeology are Food Reconstruction Using Isotopic Transferred Signals (FRUITS)^21,22^ and MixSIAR^23,24^. The MixSIAR model uses a Markov chain Monte Carlo method to derive the most likely logistic model from the probability distribution^23^. MixSIAR allows users to compare the estimated dietary compositions of two or more groups, provided all groups are directly comparable. However, isotopic mixing models can be complicated for archaeological materials due to ecological differences between modern sources and prehistoric consumers^25^. To avoid the issue between modern and ancient ecosystems, in this study, we used consumers and food sources from the Imdang burial mounds and ran MixSIAR models to estimate the proportional contribution of contemporary sources to each individual, providing an estimate of the variability in the estimated proportion.

**2. Archaeological Information**

**1) Imdang burial mounds**

The Imdang burial mounds is a well-known archaeological site located on a gentle hillside of a mountain in the middle of an alluvial plain that was formed by the Kumho River near Gyeongsan City, North Gyeongsang Province, South Korea, (35° 50’ 22” N, 128° 44’ 59” E) ^26,27^ (see Fig. 1). The burial site is situated 50-75 m above sea level and covers a wide area of approximately 35,000 m^2^ on the three districts of Imdang, Joyeung and Bujeok. The site was first excavated in 1982 by the Yeungnam University Museum (YUM) and later excavated by the Yeungnam University Museum (YUM), Yeongnam Institute of Cultural Heritage (YICH), and Korea Cultural Heritage Foundation (KCHF) until 2010^26,28-30^. Based on archaeological records and cultural features, this mortuary site was occupied during the Proto-Three Kingdoms period (BC 108 - 313 AD). This site contained approximately 1600 burials and a large amount of grave goods such as gilt-bronze crowns and ornaments, pottery, iron weapons and tools as well as human and animal bones. On the basis of the location and scale of the tombs and the lavish grave materials, it is assumed that those interred at this site belonged to the highest status within the early ancient Apdok state^27,31,32^. In particular, the excavation of a gilt-bronze crown and ornaments (belt, rings, and bracelets) which is considered a symbol of local royalty suggests that the tomb’s owners interred at Imdang represent local elites from the Gyeongsan areas^33,34^.

**2) Burial structure and human remain**

According to the archaeological reports, four different types of burials were identified inside the Imdang tombs: jar coffins: (*ongkwanmyo*), single wooden chambers: (*dankwakmyo*), double wooden chambers: (*jubukwakmyo*), and stone chambers with a horizontal entrance (*hoenggusik suksilmyo*)^32,35^. The large number of burial mounds that contain different types of burials suggests the possibility of multiple burials. Among the reported 182 graves, the double wooden chambers (*jubukwakmyo*) are the most common. The double chamber tombs were mainly comprised of a main rectangle (*jukwak*) and a square auxiliary chamber (*bukwak*) (Fig. 1)^35^. In the main chamber (*jukwak*), there was a central space for a coffin. Surrounding the coffin on all four sides, there were grave goods including ornaments and iron weapons. At the head of the coffin, a large wooden chest was installed that contained numerous ceramic containers, cups, and pedestaled vessels that were presumably filled with foods. The auxiliary burial chamber contained a larger square space for the deposition of additional grave goods and offerings such as serving pots and processed animals^35^. Furthermore, between one to five individuals were sacrificed (*sunjang*) and buried inside both the main and auxiliary chambers following the death of a tomb’s owner^34,36,37^. After the coffin, grave goods, and sacrificed individuals were deposited in the burial chamber, a large mound of boulder-sized stones was constructed above the chamber^36^. Finally, an earthen mound was constructed above the cover stones that reached 6.5 m high and 20~30 m in diameter. The mound was carefully constructed by adding layers of clay and other sediments to stabilize the tumulus. Horse tack, glass beads, and other animal bones were buried in the peak of the mound, suggesting a final ritual to mark the end of the construction of the tomb^36,38^.

The Imdang site has well preserved human skeletal remains. During the excavations, the skeletal remains of more than 500 individuals within 182 graves were recovered and 259 individuals in the Yeungnam University Museum were anthropologically examined^39,40^. The age and sex assessments of the human skeletons were undertaken in the Department of Anthropology at Seoul National University^40^. According to the osteological reports on the 259 individuals, there were 66 male and 54 female skeletons over 20 years, and 51 individuals aged less than 20 years^39,40^. Young adults between the ages of 21 and 35 were the most common (n = 64) and adults between the ages of 35-50 were next most common (n = 46). Ancient mitochondrial DNA analyses were also conducted on the human skeletal remains of 67 individuals recovered from several tombs at the Imdang site. The analyses revealed that at least four sacrificed individuals shared matrilineal affinities with the tomb’s main owner, suggesting that some individuals were related to the elites^41^.

**3) Floral and faunal remains**

Analysis of the archaeobotanical remains showed that domesticated crops were recovered in the Imdang graves. Rice grains (*Oryza sativa*) with husks were found on the floor of the chamber and inside pottery, and marks of rice were found on the surface of iron artifacts inside burials^42^. Until recently, rice was the most common crop found in most high-status burials from the Proto-Three Kingdoms period (BC 108 – 313 AD)^43,44^. This suggests that rice has been an important crop both economically and symbolically to the high-status people during this time period. Previous studies showed that rice grain size increased during the Proto-Three Kingdoms period due to the development of better agricultural methods^44-46^. Along with rice, foxtail millet (*Setaria italica*), barnyard millet (*Echinochloa crusgalli*), and perilla (*Perilla frutescens*) were also recovered from the burials^42^. These plants are believed to be important crops because they were found in other settlement sites near the Imdang cemetery^47^. Seeds of fruits such as peach (*Prunus persica*), Caucasian persimmon (*Diospyros lotus L.*), and apricot (*Prunus armeniaca*) were recovered in container jars^42^. These fruits were harvested and consumed for ritual offerings when they ripened in mid-summer.

The Imdang burials contained a variety of terrestrial and marine animals (Supplementary Fig. 1). The analysis of fauna remains from the graves showed that the most represented terrestrial animals were wild birds^48-51^. According to the zooarchaeological reports, bird specimens count for more than 605 individuals and the most common species is pheasant (*Phasianus colchicus*)^49,50^. A large variety of bird bones were exposed, and non-migratory birds such as pheasant, and migratory birds such as wild goose (*Anser*), swan (*Cygnus*), bustard (*Otis*), crane (*Grus*) and mallard (*Anas*) were frequently identified^50^ (Supplementary Figure 1). Most of the bird bones were found in container jars or pottery inside graves. In addition, terrestrial mammals were recovered from the Imdang burials. According to the faunal analysis, more than 69 terrestrial mammals were found^38,51^. This included diverse specimens of wild herbivores such as deer (*Cervus*), wild boar (*Sus scrofa*), and hare (*Lepus*). Along with wild animals, domestic animals such as dogs (*Canis familiaris*), pigs (*Sus domesticus*), cattle (*Bos taurus*), and horses (*Equus caballus*) were found on the capstone and stone slabs of the graves^38^. It was believed that the horse was one of the main mammal used for animal sacrifice during the Proto-Three Kingdoms period (BC 108 – 313 AD). However, fauna evidence showed that other mammals such as dog, cattle, and pig were also used as ritual offerings in the Imdang mounds.

Although located on an alluvial plain about 60km from the coast, the Imdang burials contained a variety of marine fish and shellfish (Supplementary Figure 1)^42,52,53^. The marine fish species from the Imdang burials were sharks (*Carcharhinidae*, *Lamnidae*, *Squalidae*), amberjack (*Seriola*), sea breams (*Sparidae*), rockfish (*Sebastiscus*), flatfish (*Paralichthyidae*), and puffer fish (*Tetraodontidae*) ^42^. The most commonly found fish bones in the burials are sandbar shark (*Carcharhinidae*), white shark (*Lamnidae*), and dogfish shark (*Squalidae*). Even in today, salted shark (*Dombaeki*) is used as a ritual offering only in the same regions^54^. Imdang burials also exposed a large amount of shells of several species of clams, sea snails, abalones and scallops. The main species of shellfish are turban shell (*Turbinidae*), conch (*Turritellidae*), abalone (*Haliotidae*), clam (*Veneridae*), and oyster (*Ostreidae*) ^42,52^. The marine fish and shellfish recovered from the burials indicated that they were transported inland from the east and south coast of the Korean Peninsula. Unlike other burials from the Three-Kingdoms periods, the Imdang burials also contained freshwater fish such as carp (*Cyprinidae*) and freshwater snail (*Viviparidae*)^42^. It is assumed that these freshwater species were also harvested from the creeks and rivers next to the burials.

**References**

1. Schwarcz, H. & Schoeninger, M. Stable isotope analyses in human nutritional ecology. *Yearb. Phys. Anthropol.* **34**, 283–321 (1991).

2. Katzenberg, M.A. in *Biological anthropology of the human skeleton.* (eds. Katzenberg, M.A. & Saunders S.R.) 305–327 (New York: Wiley-Liss. 2000).

3. Richards, M.P. & Hedges, R.E.M. Stable isotope analysis reveals variations in human diet at the Poundbury Camp cemetery site. *J. Archaeol. Sci.* **25**, 1247–1252. (1998).

4. Yoder, C. Let them eat cake? Status-based differences in diet in medieval Denmark. *J. Archaeol. Sci.* **39**, 1183-1193 (2012).

5. Ma, Y. *et al.* [Isotopic perspectives (δ^13^C, δ^15^N, δ^34^S) of diet, social complexity, and animal husbandry during the proto-shang period (ca. 2000–1600 BC) of China](http://onlinelibrary.wiley.com/doi/10.1002/ajpa.22980/abstract). *Am. J. Phys. Anthropol.* **160**, 433–445 (2016).

6. Van der Merwe, N.J. Carbon isotopes, photosynthesis, and archaeology. *Am. Sci.* **70**, 596–606 (1982).

7. Krueger, H.W., Sullivan, C.H. Models for carbon isotope fractionation between diet and bone. Stable isotope in nutrition, ACS symposium. Washington, DC: *ACS Symp Ser Am Chem Soc.* 205–220 (1984).

8. DeNiro, M.J. & Epstein, S. Influence of diet on the distribution of carbon isotopes in animals. *Geochim. Cosmochim. Acta* **42**, 495–505 1978.

9. Hu, Y., Ambrose, S.H., &Wang, C. Stable isotopic analysis of human bones from Jiahu site, Henan, China: implications for the transition to agriculture. *J. Archaeol. Sci.* **33**,1319–1330 (2006).

10. Wang, T. *et al.* Tianshanbeilu and the Isotopic Millet Road: Reviewing the late Neolithic/Bronze Age radiation of human millet consumption from north China to Europe. *Natl. Sci. Rev.* **6**, 1024-1039 (2019).

11. Choy, K. *et al.* Direct isotopic evidence for human millet consumption in the Middle Mumun period: implication and importance of millets in early agriculture on the Korean Peninsula. *J. Archaeol. Sci.* **129**, 105372 (2021).

12. Bocherens, H. & Drucker, D. Trophic level isotopic enrichment of carbon and nitrogen in bone collagen: case studies from recent and ancient terrestrial ecosystems. *Int. J. Osteoarcheol.* **13**, 46–53 (2003).

13. Hedges, R.E.M. & Reynard, L.M. Nitrogen isotopes and the trophic level of humans in archaeology. *J. Archaeol. Sci.* **34**,1240–1251 (2007).

14. Lee-Thorp, J.A. On isotopes and old bones. *Archaeometry* **50**, 925–950. (2008).

15. Richards, M.P. in *Archaeological Science: An Introduction.* (eds. Richards, M.P., Britton, K.) 125–145. (Cambridge University Press, Cambridge, 2020).

16. Newsome, S.D. *et al.* Dietary reconstruction of an early to middle Holocene human population from the central California coast: insights from advanced stable isotope mixing models. *J. Archaeol. Sci.* **31**, 11010-1115 (2004).

17. Halffman, C.M. et al. Ancient Beringian paleodiets revealed through multiproxy stable isotope analyses. *Sci. Adv.* **6**, eabc1968 (2020).

18. Cheung, C. & Szpak, P. Interpreting past human diets using stable isotope mixing models. *J. Archaeol. Method Theory* <https://doi.org/10.1007/s10816-020-09492-5> (2020).19. Haines, E. B. Relation between the stable carbon isotope composition of fiddler crabs, plants, and soils in a salt marsh. *Limnol. Oceanogr.* **21**, 880–883 (1976).

20. McConnaughey, T. & McRoy, C. P. Food-web structure and the fractionation of carbon isotopes in the Bering Sea. *Mar. Biol*. **53**, 257–262 (1979).

21. Fernandes, R., Millard, A., Brabec, M., Nadeau, M., Grootes, P. Food reconstruction using isotopic transferred signals (FRUITS): A Bayesian model for diet reconstruction. *PLoS One*, **9**, e87436 (2014).

22. Fernandes, R., Grootes, P., Nadeau, M.J. & Nehlich, O. Quantitative diet reconstruction of a Neolithic population using a Bayesian mixing model (FRUITS): The case study of Ostorf (Germany). *Am. J. Phys. Anthropol.* **158**, 325–340 (2015).

23. Stock, B. *et al.* Analyzing mixing systems using a new generation of Bayesian tracer mixing models. *PeerJ.* 6, e5096 (2018).

24. Stock, B. & Semmens, B. MixSIAR GUI User Manual. Version 3.1. https://github.com/brianstock/ MixSIAR (2016).

25. Richards, M. P. *et al.* Marine diets in the European late Upper Paleolithic: a reply to Bocherens and Drucker. *J. Hum. Evol.* **51**, 443–444 (2006).

26. Yeungnam University Museum (YUM). Excavation reports on Imdang burial mounds-Imdang 2ho (2002).

27. Lee, H.J. Evolution and transformation of the ancient local polity in Gyeongsan area. *J. Yeungnam Archaeol.* **34**, 5-34 (2004).

28. Yeungnam Institute of Cultural Properties (YICH). *Excavation reports on Imdang burial mounds Ⅰ* (1999).

29. Yeungnam Institute of Cultural Properties (YICH). *Excavation reports on Imdang burial mounds Ⅱ ~ Ⅳ* (2001).

30. Korea Cultural Heritage Foundation (KCHF). Gyeongsan Imdang site Ⅰ ~ Ⅵ and Appendix, Excavation Reports Book 5. (1998).

31. Kim, Y.S. The ancient tombs in Imdang area. *Yeungnam Archaeol. Rev.***13**, 207-221 (2004).

32. Jang, Y.S. Establishment and change of the ancient polity of Gyeongsan area researched through the Imdang site*. J. Korean Field Archaeol.* **3**, 44-85 (2007).

33. Kim, D.W. in *A Study on the Funeral System of Ancient Tombs at the Im-dang Site.* Unpublished Ph.D. dissertation. (Yeungnam University: Gyeongsan, 2014).

34. Kim, D.W. The possibility of non-burial of bodies on tombs of the province under Silla through burial of Gyeongsan Joyeung E-Ⅱ-2. *Sogang J. Early Korean Hist.* **28**, 395-422. (2018).

35. Choi, B.I. A study on the shape of main burial chamber and accessory burial chamber in Imdang, Gyeongsan. *Sogang J. Early Korean Hist.* **22**, 349-378 (2016).

36. Conte, M. & Kim, J. An economy of human sacrifice: the practice of *sunjang* in an ancient state of Korea. *J. Anthropol. Archaeol.* **44**,14-30 (2016).

37. Kim, D.W. A study on the characteristics of buried person and the culture of sacrificial burial of ancient tombs in Imdang. *Sogang J. Early Korean Hist.* **20**, 147-183. (2015).

38. Ko, E.B. Animal sacrifice rituals in ancient Korea- with focus on Silla tombs of the Three Kingdoms period. *Anat. Biol. Anthropol.* **33**, 69-77 (2020).

39. Jung, S.S. & Choi, B.I. A study on the average lifespan of the tomb owner from Yimdang site, Gyeongsan and Yeanri site, Gimhae. *J. Korean Field Archaeol.* **22**, 27-52 (2015).

40. Yeungnam University Museum (YUM). *Archaeological reports on the human bones from Imdang site in Gyeongsan* (2013).

41. Ha, D.R. A study on the status of the victims of the Silla sacrificial burials at Imdang. *J. Korean Archaeol. Soc.* **79**, 175–204 (2011).

42. Yeungnam University Museum (YUM). *Archaeological reports on the animal remains from Imdang site in Gyeongsan Ⅱ fish and shellfish* (2018).

43. Ahn, S.M. Crops and fruits in South Korea during the 1^st^ century BC~ AD 4^th^ century, *Agriculture in Korean Archaeology, the Conference of Korean Archaeology*. 69-138 (2013).

44. Kim, M. 2015. Rice in ancient Korea: status symbol or community food? *Antiquity* **89**, 838–853 (2015).

45. Jeong, Y. Rice cultivation during the Proto-Three-Kingdoms period in Korea. *J. Korean Anc. Hist. Soc.* **69**, 19–38 (2010).

46. Kim, M., Ahn, S.M. & Jeong, Y. Rice (Oryza sativa L.): Seed-size comparison and cultivation in ancient Korea. *Econ. Bot.* **67**, 378–386. (2013).

47. Daegu National Museum. in *Life and death of Apdok people*. (2000).

48. Ko, E.B. in *Imdang Cemetery, Gyeongsan X*. (ed. Yeungnam University Museum), 133–135 (Museum, Gyeongsan, Korea, 2013).

49. Ko, E.B. Seventy Feetless Birds. *the 41^st^ conference of the Korean Archaeological Society*. 186-96 (2017).

50. Ko, E.B. Birds for the Dead: animal offerings from high-mound tombs at Imdang. *J. Korean Archaeol. Soc.* **106**, 4-41 (2018).

51. Yeungnam University Museum (YUM). *Archaeological reports on the animal remains from Imdang site in Gyeongsan Ⅰ- mammals and birds* (2017).

52. Kim, E.Y. in *The King of Apdok-guk, Dreaming of His Immortality.* (ed. Hwang, J.H.) 192–204 (Gyeongsan Museum: Gyeongsan, 2011).

53. Ko, E.B. in *The King of Apdok-guk, Dreaming of His Immortality.* (ed. Hwang, J.H.) 205–208 (Gyeongsan Museum: Gyeongsan, 2011)

54. Kim, E.O. *et al.* Physicochemical comparison of two different shark meats used for preparation of Dombaeki. *Korean J. Food Preserv.* **15**, 711-718. (2008).

55. Shin, J.Y. & Lee, J.J. Dietary reconstruction of human remains from the Gyeongsan Imdang-dong burials using stable carbon and nitrogen isotope analysis. *J. Korean Archaeol.* **70**, 84–108. (2009).

**
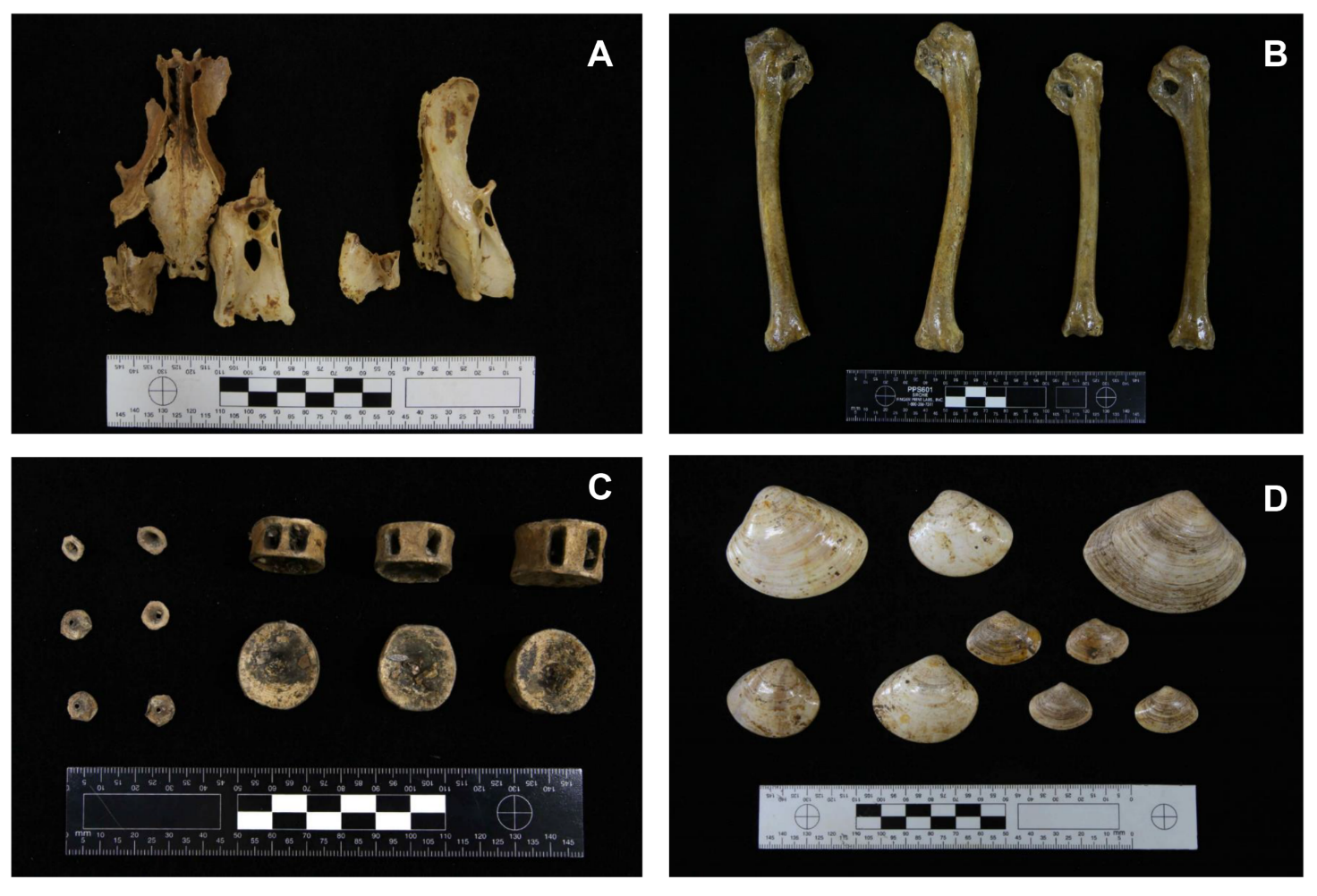
**

**Supplementary Figure 1.** Examples of animal remains from the Imdang burial mounds: pelvis and sacrum of pheasant (A), humerus of wild goose (B), vertebrate of sandbar sharks (C), and shells of clams (D) (Photo courtesy of YUM).


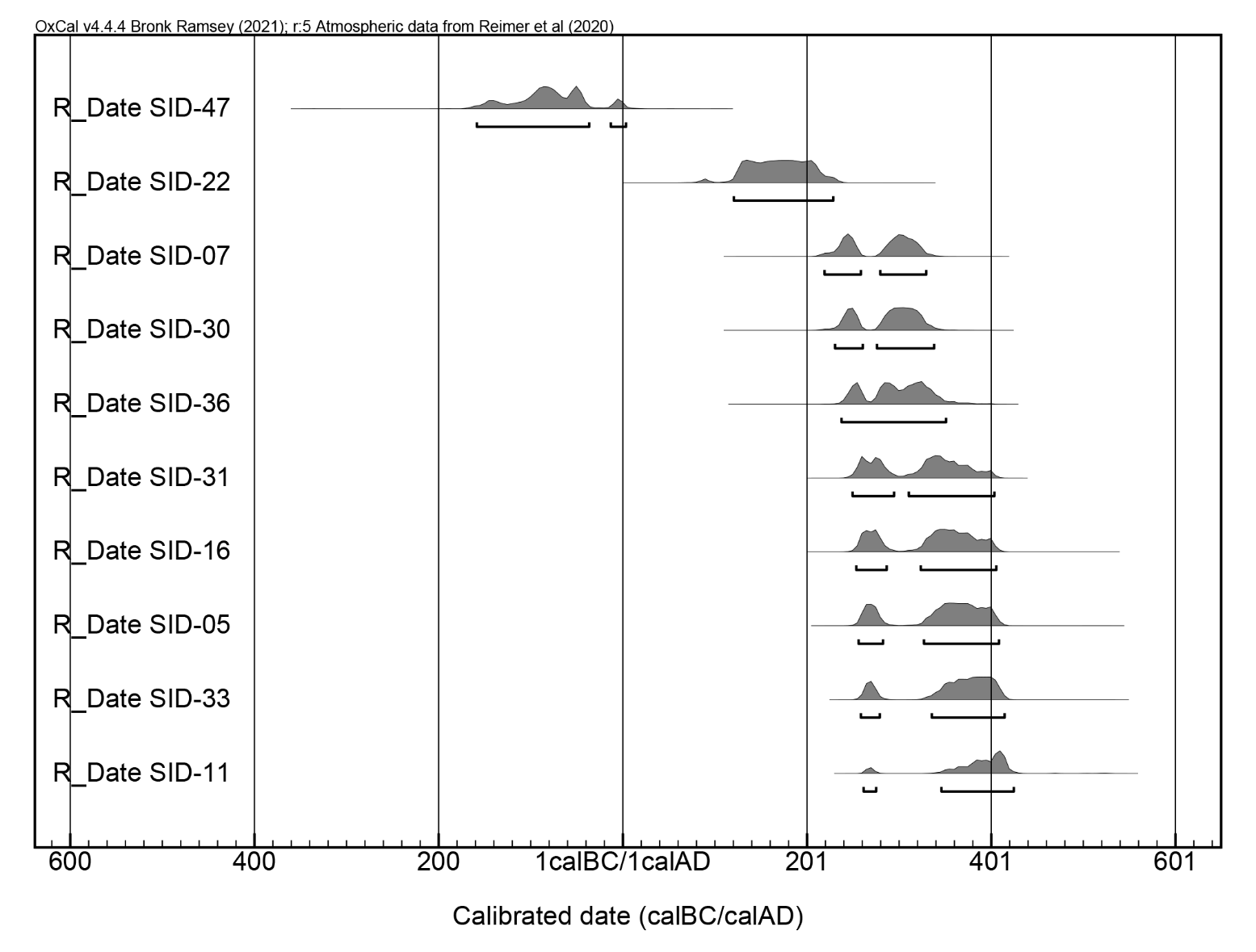


**Supplementary Figure 2.** OxCal sequence model for the calibrated radiocarbon dates obtained from Imdang humans. The radiocarbon dates prove that the Imdang cemetery was used from approximately 80 BC to 394 AD. The dates and calibrations are in Supplementary Tables 1.

**
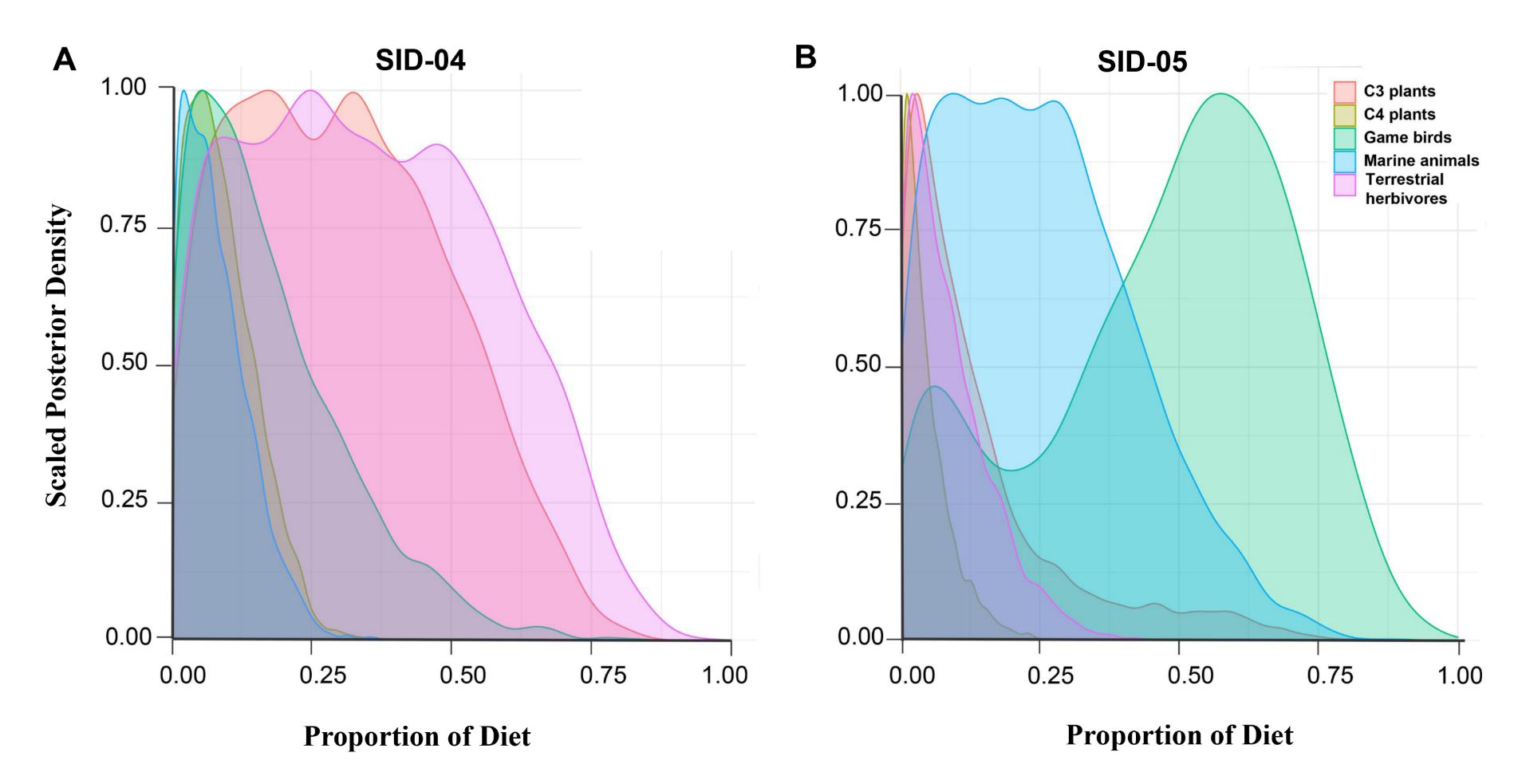
**

**Supplementary Figure 3.** Estimates of proportional source contribution to each human in the double wooden chamber tomb (Joyeung E I-2ho) generated by a MixSIAR model. There is a clear difference in source contribution between sample individual SID-04 (A) and SID-05 (B). While SID-04 has high contribution of terrestrial herbivores, SID-05 has high contribution of game birds and marine animals.

**Supplementary Table 1.** AMS radiocarbon dates on bone collagen of humans and animals from the Imdang tombs in this study.

| **Sample name** | **Location** | **Burial** | **Species** | **Element** | **C:N** | **^14^C date (BP)** | **Calendar Age Cal BC (2σ)** | **Median Age Cal BC** |
| --- | --- | --- | --- | --- | --- | --- | --- | --- |
| SID-47 | Joyeung EⅢ-29ho-1 | Single Wooden chamber | *Homo sapiens* | Femur | 3.3 | 2070 ± 20 | BC158- 3 AD | 80 BC |
| SID-22 | Joyeung EⅡ-7ho-1 | Double Wooden chamber | *Homo sapiens* | Rib | 3.3 | 1875 ± 20 | 122-228 | 170 AD |
| SID-07 | Joyeung EⅡ-1ho-1 | Stone Chamber | *Homo sapiens* | Femur | 3.2 | 1790 ± 20 | 219-329 | 293 AD |
| SID-30 | Joyeung EⅢ-3ho-2 | Double Wooden chamber | *Homo sapiens* | Limb | 3.2 | 1780 ± 20 | 232-338 | 296 AD |
| SID-36 | Joyeung EⅢ-8ho-1 | Double Wooden chamber | *Homo sapiens* | Phalanges | 3.3 | 1760 ± 20 | 239-360 | 300 AD |
| SID-31 | Joyeung EⅢ-3ho-3 | Double Wooden chamber | *Homo sapiens* | Limb | 3.2 | 1730 ± 20 | 251-404 | 335 AD |
| SID-16 | Joyeung EⅡ-3ho-2 | Double Wooden chamber | *Homo sapiens* | Cranium | 3.3 | 1720 ± 20 | 254-406 | 347 AD |
| SID-05 | Joyeung EⅠ-2ho-2 | Double Wooden chamber | *Homo sapiens* | Cranium | 3.2 | 1710 ± 20 | 257-408 | 358 AD |
| SID-33 | Joyeung EⅢ-4ho-2 | Double Wooden chamber | *Homo sapiens* | Limb | 3.3 | 1695 ± 20 | 260-414 | 372 AD |
| SID-11 | Joyeung EⅡ-2ho-3 | Double Wooden chamber | *Homo sapiens* | Rib | 3.2 | 1670 ± 20 | 236-424 | 394 AD |
| SID-15 | Joyeung EⅠ-1ho | Double Wooden chamber | *Carcharhinus plumbeus* | Vertebrate | 3.3 | 2315 ± 15 | BC 403- BC 381 | 393 BC |
| SID-18 | Imdang 2ho North | Double Wooden chamber | *Canis familiaris* | Vertebrate | 2.9 | 1775 ± 25 | 220-349 | 295 AD |

**Supplementary Table 2.** Results of stable carbon and nitrogen isotope analysis of faunal samples from the Imdang tombs in this study. NA: not available

| **Sample ID.** | **Location** | **Species** | **common name** | **Element** | **Chamber** | **Conc C** | **Conc N** | **δ^13^C** | **δ^15^N** | **C:N** |
| --- | --- | --- | --- | --- | --- | --- | --- | --- | --- | --- |
| SID-50 | Joyeung EⅠ-1 Ho | *Carcharhinus plumbeus* | Sandbar shark | Vertebrate | NA | 43.5 | 15.5 | -12.3 | 12.5 | 3.3 |
| SID-51 | Joyeung EⅠ-2 Ho | *Carcharhinus plumbeus* | Sandbar shark | Vertebrate | Auxiliary | 43.0 | 14.9 | -12.6 | 13.0 | 3.4 |
| SID-53 | Joyeung EⅡ-2 Ho | *Seriola quinqueradiata* | Amberjack | Vertebrate | Auxiliary | 51.9 | 17.5 | -12.0 | 12.1 | 3.5 |
| SID-58 | Joyeung EⅡ-3Ho | *Phasianus colchicus* | Pheasant | Femur | Auxiliary | 42.9 | 15.0 | -16.2 | 5.2 | 3.3 |
| SID-60 | Joyeung EⅡ-3Ho | *Otis tarda* | Great bustard | Tarsal | Auxiliary | 43.6 | 15.3 | -18.5 | 5.9 | 3.3 |
| SID-61 | Joyeung EⅡ-3 Ho | *Anser fabalis* | Wild goose | Humerus | Auxiliary | 45.2 | 16.3 | -22.4 | 9.8 | 3.2 |
| SID-63 | Joyeung EⅡ-4 Ho | *Cygnus columbianus* | Swan | Vertebrate | Auxiliary | 45.9 | 16.0 | -15.3 | 7.2 | 3.3 |
| SID-64 | Joyeung EⅡ-4 Ho | *Anas platyrhynchos* | Marine duck | Humerus | Auxiliary | 38.2 | 13.0 | -12.6 | 10.1 | 3.4 |
| SID-65 | Joyeung EⅡ-5 Ho | *Bos taurus* | Cattle | Metacarpal | NA | 40.4 | 14.2 | -14.0 | 6.6 | 3.3 |
| SID-67 | Imdang 2 Ho North | *Canis familiaris* | Dog | Vertebrate | Main | 44.0 | 15.3 | -16.5 | 9.1 | 3.4 |
| SID-68 | Imdang 2 Ho North | *Sus scrofa* | Wild boar | Phalange | Auxiliary | 41.4 | 13.4 | -20.4 | 2.3 | 3.6 |
| SID-69 | Joyeung EⅡ-3 Ho | *Equus caballus* | Horse | Tooth | NA | 46.3 | 16.3 | -16.7 | 4.2 | 3.3 |
| SID-70 | Imdang 6A-ho | *Sus domesticus* | Pig | Tooth | NA | 42.0 | 14.0 | -19.4 | 7.2 | 3.5 |
| SID-71 | Imdang 2 Ho North | *Lepus* | Hare | Vertebrate | Main | 45.9 | 16.1 | -21.1 | 3.1 | 3.3 |

**Supplementary Table 3**. List of human samples for bulk stable carbon and nitrogen isotope analyses in this study, NA: not available

| **Sample ID** | **Location** | **Species** | **Element** | **Burial** | **Chamber** | **Type** | **Sex** | **Age** | **Conc C** | **Conc N** | **δ^13^C** | **δ^15^N** | **C:N** |
| --- | --- | --- | --- | --- | --- | --- | --- | --- | --- | --- | --- | --- | --- |
| SID-04 | Joyeung EⅠ-2ho-1 | *Homo sapiens* | Phalange | Double Wooden chamber | Main | Retainer | NA | 6-10 | 44.0 | 15.1 | -18.0 | 7.8 | 3.4 |
| SID-05 | Joyeung EⅠ-2ho-2 | *Homo sapiens* | Cranium | Double Wooden chamber | Main | Elite | NA | 36-50 | 41.3 | 14.6 | -18.3 | 13.2 | 3.3 |
| SID-07 | Joyeung EⅡ-1ho-1 | *Homo sapiens* | Femur | Stone Chamber | Chamber | Elite | NA | Adult | 44.2 | 15.9 | -18.0 | 13.2 | 3.2 |
| SID-08 | Joyeung EⅡ-1ho-2 | *Homo sapiens* | Femur | Stone Chamber | Chamber | Retainer | NA | Adult | 44.6 | 16.0 | -17.6 | 10.0 | 3.2 |
| SID-09 | Joyeung EⅡ-2ho-1 | *Homo sapiens* | Cranium | Double Wooden chamber | Main | Retainer | NA | 15-18 | 44.1 | 14.1 | -19.5 | 9.6 | 3.6 |
| SID-10 | Joyeung EⅡ-2ho-2 | *Homo sapiens* | Femur | Double Wooden chamber | Main | Retainer | NA | Adult | 40.6 | 14.8 | -17.5 | 10.1 | 3.2 |
| SID-11 | Joyeung EⅡ-2ho-3 | *Homo sapiens* | Rib | Double Wooden chamber | Main | Retainer | NA | 7.5-12.5 | 42.9 | 15.2 | -18.5 | 9.9 | 3.3 |
| SID-12 | Joyeung EⅡ-2ho-4 | *Homo sapiens* | Femur | Double Wooden chamber | Auxiliary | Elite | NA | 21-35 | 40.9 | 13.2 | -20.2 | 12.0 | 3.6 |
| SID-13 | Joyeung EⅡ-2ho-5 | *Homo sapiens* | Femur | Double Wooden chamber | Auxiliary | Retainer | M | Adult | 41.0 | 14.1 | -18.0 | 9.2 | 3.4 |
| SID-14 | Joyeung EⅡ-2ho-6 | *Homo sapiens* | Femur | Double Wooden chamber | Auxiliary | Retainer | NA | Adult | 33.0 | 10.6 | -19.4 | 9.2 | 3.6 |
| SID-15 | Joyeung EⅡ-3ho-1 | *Homo sapiens* | Fibula | Double Wooden chamber | Main | Elite | NA | 21-35 | 41.6 | 13.3 | -19.0 | 9.8 | 3.6 |
| SID-16 | Joyeung EⅡ-3ho-2 | *Homo sapiens* | Cranium | Double Wooden chamber | Main | Retainer | NA | 20 | 41.7 | 14.7 | -18.4 | 8.0 | 3.3 |
| SID-17 | Joyeung EⅡ-3ho-3 | *Homo sapiens* | Femur | Double Wooden chamber | Auxiliary | Retainer | F | Adult | 41.8 | 14.3 | -16.8 | 7.6 | 3.4 |
| SID-18 | Joyeung EⅡ-3ho-4 | *Homo sapiens* | Fibula | Double Wooden chamber | Auxiliary | Retainer | F | 21-35 | 39.1 | 12.7 | -18.8 | 9.2 | 3.6 |
| SID-19 | Joyeung EⅡ-4ho-1 | *Homo sapiens* | Limb | Double Wooden chamber | Auxiliary | Elite | NA | NA | 42.3 | 15.6 | -18.8 | 12.3 | 3.2 |
| SID-20 | Joyeung EⅡ-5ho-1 | *Homo sapiens* | Humerus | Stone Chamber | Chamber | Elite | M | Adult | 41.1 | 15.2 | -19.6 | 12.0 | 3.2 |
| SID-21 | Joyeung EⅡ-6ho-1 | *Homo sapiens* | Rib | Double Wooden chamber | Main | NA | F | 21-35 | 38.1 | 13.6 | -18.9 | 10.7 | 3.3 |
| SID-22 | Joyeung EⅡ-7ho-1 | *Homo sapiens* | Rib | Double Wooden chamber | Main | Elite | M | 21-40 | 45.0 | 14.5 | -19.8 | 12.4 | 3.6 |
| SID-25 | Joyeung EⅢ-2ho-2 | *Homo sapiens* | Rib | Double Wooden chamber | Main | Retainer | M | 21-35 | 39.7 | 14.7 | -17.7 | 10.1 | 3.2 |
| SID-26 | Joyeung EⅢ-2ho-3 | *Homo sapiens* | Rib | Double Wooden chamber | Main | Retainer | NA | 6-12 | 36.9 | 13.6 | -17.3 | 8.1 | 3.2 |
| SID-27 | Joyeung EⅢ-2ho-4 | *Homo sapiens* | Limb | Double Wooden chamber | Auxiliary | Retainer | F | 21-35 | 39.3 | 14.9 | -18.0 | 9.6 | 3.1 |
| SID-28 | Joyeung EⅢ-2 ho-5 | *Homo sapiens* | Limb | Double Wooden chamber | Auxiliary | Retainer | M | 21-35 | 41.0 | 15.7 | -17.4 | 10.1 | 3.1 |
| SID-29 | Joyeung EⅢ-3ho-1 | *Homo sapiens* | Rib | Double Wooden chamber | Main | Elite | NA | Adult | 43.3 | 15.1 | -18.6 | 12.6 | 3.4 |
| SID-30 | Joyeung EⅢ-3 ho-2 | *Homo sapiens* | Limb | Double Wooden chamber | Main | Retainer | F | Adult | 43.6 | 15.2 | -18.7 | 11.8 | 3.3 |
| SID-31 | Joyeung EⅢ-3 ho-3 | *Homo sapiens* | Limb | Double Wooden chamber | Auxiliary | Retainer | M | 21-35 | 42.5 | 14.9 | -17.8 | 10.8 | 3.3 |
| SID-33 | Joyeung EⅢ-4 ho-2 | *Homo sapiens* | Limb | Double Wooden chamber | Auxiliary | Retainer | F | 31-50 | 42.0 | 14.6 | -17.0 | 9.0 | 3.4 |
| SID-36 | Joyeung EⅢ-8 ho-1 | *Homo sapiens* | Phalange | Double Wooden chamber | Main | Elite | NA | 3-5 | 43.7 | 14.4 | -18.1 | 9.8 | 3.5 |
| SID-37 | Joyeung EⅢ-8 Ho-2 | *Homo sapiens* | Rib | Double Wooden chamber | Main | Retainer | NA | 15 | 47.1 | 15.8 | -17.8 | 9.9 | 3.5 |
| SID-46 | Joyeung EⅢ-26 ho-1 | *Homo sapiens* | Limb | Single Wooden chamber | NA | NA | NA | 21-35 | 41.3 | 13.8 | -19.7 | 11.0 | 3.5 |
| SID-47 | Joyeung EⅢ-29 ho-1 | *Homo sapiens* | Femur | Single Wooden chamber | NA | NA | M | Adult | 39.0 | 12.5 | -19.9 | 10.4 | 3.6 |

**Supplementary Table 4.** MixSIAR model estimates for Imdang humans in proportional contribution of four food sources with Bayesian Credibility Intervals (5% and 95%). The previously published isotopic values of humans from Joyeung C (CJI-1~ CJⅡ-28) are also added and calculated using MixSIAR^55^.

|  |  | **C_3_ plants** |  |  |  | **Terrestrial herbivores** |  |  |  | **Game birds** |  |  |  | **Marine animals** |  |  |  | **C_4_ plants** |  |
| --- | --- | --- | --- | --- | --- | --- | --- | --- | --- | --- | --- | --- | --- | --- | --- | --- | --- | --- | --- |
| **Sample ID** | Mean% | 5% Cl | 95% Cl |  | Mean% | 5% Cl | 95% Cl |  | Mean% | 5% Cl | 95% Cl |  | Mean% | 5% Cl | 95% Cl |  | Mean% | 5% Cl | 95% Cl |
| SID-04 | 30 | 3 | 62 |  | 35 | 3 | 71 |  | 18 | 1 | 45 |  | 8 | 1 | 19 |  | 9 | 1 | 21 |
| SID-05 | 15 | 1 | 53 |  | 9 | 1 | 23 |  | 47 | 4 | 79 |  | 25 | 2 | 55 |  | 5 | 0 | 14 |
| SID-07 | 14 | 1 | 48 |  | 9 | 1 | 25 |  | 45 | 4 | 78 |  | 27 | 3 | 55 |  | 5 | 0 | 14 |
| SID-08 | 25 | 2 | 55 |  | 23 | 2 | 55 |  | 28 | 2 | 62 |  | 17 | 3 | 32 |  | 7 | 1 | 19 |
| SID-09 | 33 | 3 | 70 |  | 20 | 2 | 50 |  | 33 | 3 | 67 |  | 9 | 1 | 22 |  | 5 | 0 | 14 |
| SID-10 | 24 | 2 | 54 |  | 23 | 2 | 54 |  | 28 | 2 | 62 |  | 18 | 3 | 34 |  | 7 | 1 | 19 |
| SID-11 | 28 | 2 | 61 |  | 22 | 2 | 52 |  | 31 | 3 | 65 |  | 13 | 1 | 28 |  | 6 | 0 | 17 |
| SID-12 | 18 | 1 | 69 |  | 9 | 1 | 24 |  | 55 | 7 | 82 |  | 13 | 1 | 30 |  | 5 | 0 | 14 |
| SID-13 | 27 | 2 | 58 |  | 29 | 2 | 63 |  | 25 | 2 | 56 |  | 13 | 1 | 26 |  | 7 | 1 | 19 |
| SID-14 | 33 | 3 | 69 |  | 25 | 2 | 59 |  | 29 | 2 | 64 |  | 8 | 1 | 20 |  | 5 | 0 | 15 |
| SID-15 | 30 | 2 | 66 |  | 21 | 2 | 52 |  | 32 | 3 | 66 |  | 11 | 1 | 25 |  | 6 | 0 | 15 |
| SID-16 | 31 | 3 | 64 |  | 35 | 3 | 73 |  | 18 | 1 | 47 |  | 8 | 1 | 20 |  | 7 | 1 | 18 |
| SID-17 | 28 | 3 | 56 |  | 30 | 3 | 64 |  | 18 | 1 | 46 |  | 9 | 1 | 22 |  | 15 | 3 | 29 |
| SID-18 | 31 | 3 | 64 |  | 27 | 2 | 63 |  | 26 | 2 | 58 |  | 10 | 1 | 23 |  | 6 | 0 | 16 |
| SID-19 | 19 | 1 | 60 |  | 10 | 1 | 26 |  | 47 | 5 | 79 |  | 19 | 2 | 42 |  | 5 | 0 | 14 |
| SID-20 | 20 | 1 | 67 |  | 10 | 1 | 27 |  | 51 | 5 | 80 |  | 15 | 1 | 34 |  | 5 | 0 | 15 |
| SID-21 | 27 | 2 | 66 |  | 15 | 1 | 39 |  | 39 | 4 | 72 |  | 13 | 1 | 30 |  | 6 | 0 | 16 |
| SID-22 | 17 | 1 | 66 |  | 9 | 1 | 25 |  | 55 | 7 | 81 |  | 14 | 1 | 35 |  | 5 | 0 | 14 |
| SID-25 | 25 | 2 | 55 |  | 22 | 2 | 53 |  | 29 | 2 | 63 |  | 17 | 2 | 33 |  | 7 | 1 | 19 |
| SID-26 | 27 | 3 | 57 |  | 32 | 3 | 66 |  | 19 | 1 | 48 |  | 10 | 1 | 24 |  | 12 | 1 | 24 |
| SID-27 | 27 | 2 | 58 |  | 25 | 2 | 58 |  | 27 | 2 | 62 |  | 14 | 2 | 29 |  | 7 | 1 | 19 |
| SID-28 | 24 | 2 | 54 |  | 23 | 2 | 54 |  | 27 | 2 | 61 |  | 19 | 3 | 35 |  | 8 | 0 | 20 |
| SID-29 | 17 | 1 | 59 |  | 9 | 1 | 26 |  | 48 | 5 | 80 |  | 21 | 2 | 46 |  | 5 | 0 | 15 |
| SID-30 | 21 | 1 | 61 |  | 11 | 1 | 30 |  | 45 | 5 | 75 |  | 18 | 2 | 39 |  | 5 | 0 | 15 |
| SID-31 | 24 | 2 | 57 |  | 17 | 1 | 43 |  | 33 | 3 | 67 |  | 19 | 3 | 37 |  | 6 | 0 | 17 |
| SID-33 | 24 | 3 | 53 |  | 28 | 3 | 59 |  | 23 | 2 | 53 |  | 14 | 2 | 29 |  | 11 | 1 | 24 |
| SID-36 | 27 | 2 | 59 |  | 23 | 2 | 56 |  | 29 | 3 | 62 |  | 14 | 2 | 30 |  | 7 | 0 | 18 |
| SID-37 | 26 | 2 | 58 |  | 23 | 2 | 54 |  | 28 | 2 | 61 |  | 16 | 2 | 31 |  | 7 | 1 | 19 |
| SID-46 | 26 | 1 | 71 |  | 13 | 1 | 34 |  | 44 | 4 | 76 |  | 12 | 1 | 28 |  | 5 | 0 | 14 |
| SID-47 | 29 | 2 | 71 |  | 15 | 1 | 37 |  | 42 | 4 | 74 |  | 10 | 1 | 23 |  | 5 | 0 | 15 |
| CJI-11 | 15 | 1 | 51 |  | 9 | 1 | 24 |  | 46 | 4 | 78 |  | 26 | 2 | 54 |  | 5 | 0 | 14 |
| CJI-12 | 23 | 1 | 63 |  | 12 | 1 | 32 |  | 42 | 3 | 76 |  | 17 | 2 | 37 |  | 5 | 0 | 15 |
| CJI-13 | 27 | 2 | 60 |  | 25 | 2 | 58 |  | 27 | 3 | 61 |  | 14 | 2 | 29 |  | 7 | 1 | 18 |
| CJI-21 | 22 | 2 | 48 |  | 25 | 2 | 54 |  | 24 | 2 | 55 |  | 18 | 2 | 34 |  | 12 | 1 | 27 |
| CJI-22 | 23 | 2 | 51 |  | 25 | 2 | 55 |  | 24 | 2 | 57 |  | 19 | 3 | 35 |  | 10 | 1 | 24 |
| CJI-23 | 24 | 2 | 55 |  | 19 | 1 | 47 |  | 32 | 3 | 67 |  | 19 | 3 | 36 |  | 7 | 0 | 19 |
| CJI-24 | 25 | 2 | 54 |  | 31 | 3 | 62 |  | 21 | 2 | 50 |  | 12 | 1 | 26 |  | 12 | 1 | 26 |
| CJII-11 | 14 | 1 | 49 |  | 9 | 1 | 24 |  | 46 | 4 | 78 |  | 26 | 3 | 57 |  | 5 | 0 | 14 |
| CJII-13 | 24 | 2 | 54 |  | 19 | 2 | 46 |  | 31 | 4 | 64 |  | 20 | 3 | 37 |  | 7 | 0 | 18 |
| CJII-14 | 23 | 1 | 54 |  | 19 | 1 | 46 |  | 32 | 3 | 66 |  | 19 | 3 | 37 |  | 7 | 0 | 18 |
| CJII-16 | 21 | 2 | 47 |  | 21 | 2 | 48 |  | 27 | 2 | 60 |  | 23 | 5 | 41 |  | 9 | 1 | 22 |
| CJII-17 | 22 | 2 | 49 |  | 26 | 2 | 55 |  | 22 | 2 | 54 |  | 15 | 2 | 31 |  | 15 | 2 | 30 |
| CJII-22 | 27 | 2 | 58 |  | 28 | 3 | 62 |  | 25 | 2 | 58 |  | 13 | 1 | 27 |  | 7 | 1 | 19 |
| CJII-23 | 25 | 3 | 53 |  | 28 | 3 | 59 |  | 24 | 2 | 55 |  | 14 | 2 | 29 |  | 9 | 1 | 22 |
| CJII-25 | 22 | 2 | 51 |  | 22 | 2 | 52 |  | 27 | 2 | 61 |  | 20 | 3 | 38 |  | 8 | 1 | 21 |
| CJII-26 | 25 | 2 | 58 |  | 20 | 1 | 51 |  | 32 | 3 | 65 |  | 16 | 2 | 32 |  | 6 | 0 | 18 |
| CJII-27 | 25 | 2 | 59 |  | 18 | 1 | 45 |  | 34 | 3 | 67 |  | 17 | 2 | 35 |  | 6 | 0 | 17 |
| CJII-28 | 23 | 2 | 51 |  | 25 | 2 | 56 |  | 26 | 2 | 57 |  | 17 | 3 | 33 |  | 9 | 1 | 22 |
